# Characterization and Agromorphological Variation in 27 Accessions of *Chenopodium quinoa* Within the Arid Coastal Zone of Peru

**DOI:** 10.3390/plants15081147

**Published:** 2026-04-08

**Authors:** Lady E. Checmapocco-Conza, Fredy L. Huamani-Aymara, Alberto Anculle-Arenas, José L. Bustamante-Muñoz, Eric N. Jellen, Mayela Elizabeth Mayta-Anco

**Affiliations:** 1 Facultad de Agronomía, Universidad Nacional de San Agustín de Arequipa, Arequipa 04001, Peru; lchecmapocco@unsa.edu.pe (L.E.C.-C.); fhuamania@unsa.edu.pe (F.L.H.-A.); lanculle@unsa.edu.pe (A.A.-A.); jbustamantem@unsa.edu.pe (J.L.B.-M.); 2 Department of Plant and Wildlife Sciences, College of Life Sciences, Brigham Young University, Provo, UT 84602, USA; rick_jellen@byu.edu

**Keywords:** quinua, agromorphological characterization, agromorphological evaluation, selection index, multivariate analysis

## Abstract

Quinoa is an Andean crop with wide genetic variability, including the capacity to adapt to various environmental conditions, which is essential for improving its yield and quality. The present work sought to characterize and agromorphologically evaluate 27 accessions of quinoa and the commercial cultivar ‘Salcedo INIA’ (SAL) for 28 qualitative and 25 quantitative variables. The results show that, on average, maturity occurred at 120 days after sowing (DAS), with a range of 105 DAS (ACC 50) to 132 DAS (ACC 35, ACC 37, ACC 43 and SAL). Grain diameter varied between 2.39 and 1.92 mm, with ACC 29 and the SAL control having the largest seed. The percentage of saponin varied between 0.210 and 0.089%, with ACC 43 having the lowest percentage. The severity of mildew infection varied between 17.22% and 1.22%, with ACC 50 being the most resistant genotype. Grain yield ranged from 5.60 (ACC 33) to 2.44 (ACC 42) t ha^−1^. Genotypes ACC 29 and ACC 50 had the highest selection index (SI) values, at 1.10 and 1.01, respectively, being notable for their earliness, short stature, low saponin content, and seed productivity.

## 1. Introduction

Quinoa (*Chenopodium quinoa* Willd.) is an annual forb and seed crop that was domesticated for its robustness in the Andean regions of Peru and Bolivia [1], mainly in the Altiplano, where the inhabitants practice a unique form of agriculture [2]. Quinoa belongs to the Class *Magnoliopsida* and the Family *Amaranthaceae* (or *Chenopodiaceae*-*Amaranthaceae*) [3]. Quinoa stands out for its elevated vitamin, protein, essential amino acid, fiber, lipid, and mineral nutrient contents [4]. These attributes make it an important crop for alleviating hunger and promoting food security [5], and its consumption is consequently spreading throughout the world [6]. Quinoa is consumed for breakfast, lunch and dinner as a whole grain or is processed in different products [7]. Additionally, certain medicinal properties are attributed to it within its center of domestication in the Andean region [8].

The plant is able to tolerate extreme environmental conditions (salinity, cold, intense solar radiation, and drought) [9]. Its remarkable agronomic adaptation to adverse climatic conditions makes it suitable for production in regions susceptible to the effects of climate change [10]. However, that does not make the plant immune to various pests or pathogens. Downy mildew, caused by *Peronospora variabilis* Gaum, is considered the main disease in quinoa [11] and it can cause yield losses of up to 90% [12]. Diseases of secondary importance include chupadera (*Rhizoctonia* sp., *Fusarium* sp., *Phyum* sp., *Scleroum* rolfsii), brown rot (*Phoma exigua* var. *fov*) and leaf blotch (*Ascochyta hyalospora*) [13].

With the passage of time, the various quinoa strains have been exposed to the extreme environment, generating a large number of genotypes adapted to different local conditions, thus amplifying quinoa’s genetic diversity [14]. It is important to identify the agronomic characteristics that have been shaped by these edapho-climactic conditions [15]. The understanding of genetic variability plays an essential role in the genetic improvement of plant species [16].

Diverse research efforts have demonstrated the wide genetic diversity of quinoa [17], including those performed by [18] while studying agronomic and morphological characteristics in 10 genotypes from diverse regions. They observed ample genetic variability for panicle color and diameter. Similarly, ref. [19] investigated harvest and postharvest variables in 12 red quinoa lines (*Chenopodium quinoa* Willd.) selected for their mildew resistance (*Peronospora variabilis*), and found that the quantitative variables were significantly different among lines. They determined that taller plants were more highly developed, had higher quality grain, and had a higher percentage of medium-sized grain, with highly significant differences for processing characteristics and higher seed quality. Another group of researchers [14] carried out a morphological characterization of 19 quinoa varieties using 27 morphological descriptors and measured the highest coefficients of variation for quantitative characters. In addition, others [20] have evaluated selection indices based on yield components and morphological descriptors, identifying four accessions as potential progenitors for quinoa breeding programs in Colombia.

As much of the genetic diversity in cultivated quinoa exists within relatively unimproved, heterogeneous landrace accessions, phenotypic characterization of this diversity is a challenging but essential prerequisite when these genetic materials are to provide the foundation for a modern breeding program—in this case, for a low- and intermediate-elevation program breeding quinoa varieties for the hyper-arid Peruvian coastal desert at the National University of San Agustín—Arequipa. Andean quinoa producers and breeders are familiar with apparent transposon-mediated variegation in varieties such as ‘Sayaña’, with whole-genome sequencing having confirmed that transposons comprise over 55% of the genome in a single plant sequenced from the Andean ‘Real’ landrace [21]. It is therefore reasonable to assume that a significant proportion of the phenotypic variability and instability for which Andean quinoa landraces are notorious, and which can complicate line purification from these materials, is due to quinoa’s dynamic genome.

Within this paradigm, the objective of the current research work was to characterize the phenology and morphology, agronomic traits, grain quality characteristics, and selection index of 27 quinoa accessions, the end goal being to provide information on potential adaptation to the irrigated desert production area of Majes, on the Peruvian coast.

## 2. Results

### 2.1. Quantitative Phenological Variables

The results shown in Table 1 reveal that emergence was between 5 and 7 DAS for the accessions evaluated (CV = 13.60%). In the formation of the flower bud (DFBF), accessions ranged from 30 to 37 DAS (CV = 5.73%). Days at onset of flowering (DSF) averaged 53 DAS (CV = 5.62%), with accessions ranging from 42 DAS (ACC 50) to 57 DAS (ACC 43). Values for the 50% flowering stage (D50F) averaged 56 DAS (CV = 5.35%), with accessions varying from 45 DAS (ACC 50) to the control (SAL) at 62 DAS. For days to flowering termination (DEF), the range was from 51 DAS for ACC 50 to the SAL control at 70 DAS (CV = 6.10%). For days to the milky grain (DMS) stage, an average of 79 DAS was obtained, with ACC 50 achieving it at 67 DAS. For days to the pasty grain (DDS) stage, the average was 92 DAS, with ACC 29 having the shortest time of 86 DAS and the control SAL reaching DDS at 103 DAS. For number of days to 50% physiological maturity (D50PM), ACC 50 achieved the shortest time at 95 DAS, while ACC 43 and ACC 47 matured the latest at 121 DAS.

### 2.2. Emergence (EP)

**Figure 1:**
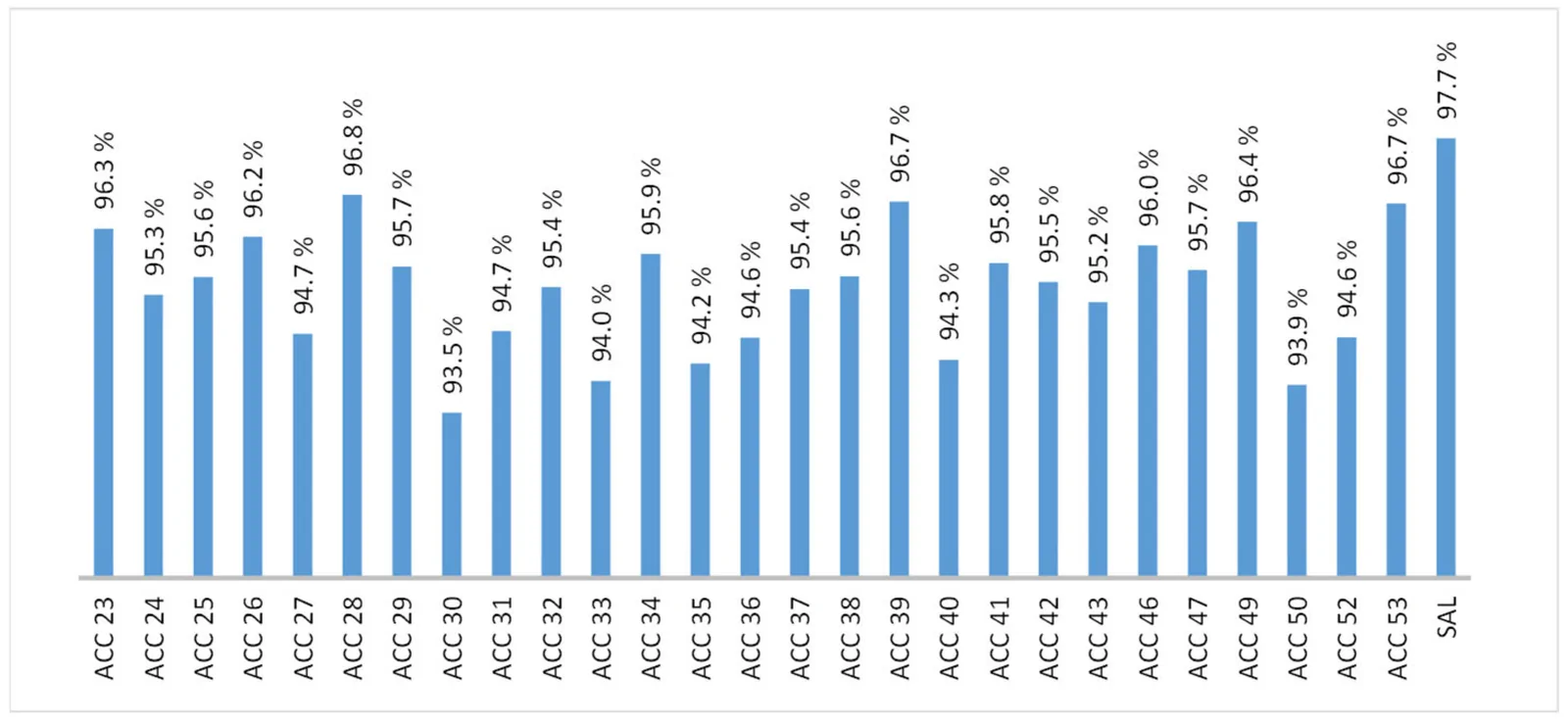
Emergence values for the 27 accessions and commercial check (SAL).

The emergence percentages of the 27 accessions and commercial control (SAL) are presented in detail in Figure 1. The average emergence value was 94.23%, with values ranging from 93.50% (ACC 30) to 97.65% (SAL). The emergence percentage could be measured by evaluating an area of 1 m^2^ randomly selected from within the middle of a plot [22].

### 2.3. Quantitative Morphological Variables

Quantitative morphological variables are presented in Table 2. For plant height (PH) at physiological maturity, we observed that ACC 43 (231.1 cm) stood out for its tall stature, statistically similar to that measured for SAL (231.1 cm), while the shortest genotype was ACC 50 (145.5 cm). For the main stem diameter (MSD) the thickest genotype was ACC 53 (13.49 mm), which was statistically similar to the check SAL (13.59 mm), while those with the thinnest stems were the statistically similar ACC 50 (10.65 mm) and ACC 47 (10.62 mm). For petiole length (PEL), the longest was ACC 38 (6.09 cm) and the shortest was ACC 41 (4.50 cm). Similarly, for leaf length (MLL), the genotype with the greatest value was ACC 38 (8.32 cm), while ACC 50 (5.76 cm) obtained the lowest value. For leaf width (MLW), ACC 52 had the greatest value (8.23 cm) and ACC 50 had the narrowest leaves (5.57 cm). For panicle length (PL), ACC 24 had the longest panicles (81.9 cm), while the check SAL had the shortest (38.9 cm), the average being 69.32 cm for the 28 strains evaluated. With respect to panicle diameter (PW), ACC 28 had the widest ones (18.77 cm) and ACC 50 had the thinnest (6.80 cm). For mildew infection (DS), ACC 43 had the most severe infection (17.22%) while ACC 50 was the most resistant to the fungus (1.22%). Line ACC 36 had the greatest specific leaf area (SLA, 923 cm^2^ g^−1^) while ACC 37 had the lowest (599 cm^2^ g^−1^). In addition, line ACC 53 had the highest number of leaf serrations (NTB, 35) while ACC 50 had the least (18).

### 2.4. Quantitative Grain Variables

Quantitative grain yield variables are shown in Table 3. With respect to grain diameter (GW), the check variety SAL (2.39 mm) and ACC 29 (2.29 mm) had significantly larger seeds than the other lines, whereas ACC 42 had the smallest seeds (1.92 mm). For seed thickness (GT), on the other hand, ACC 50 was the thickest (1.23 mm) and ACC 27 had the thinnest seed (0.93 mm). In continuation, the check variety SAL reached 3.96 g per thousand seed (G1000W), which was statistically superior to the other accessions. For hectoliter weight (GHW), ACC 31 was the heaviest (0.72 g cm^−3^). For saponin percentage (SC), ACC 25 produced the most saponin (0.210%) while ACC 43 had the least (0.08%). Accession ACC 50’s harvest index (HI) value of 33.07% was highest of all lines. In terms of seed yield (SY) ACC 33 was the highest yielding at 5.6 t ha^−1^ while ACC 42 had the lowest yield at 2.44 t ha^−1^.

### 2.5. Qualitative Morphological Variables

Qualitative morphological variables are presented in Table 4. With respect to growth habit (GH), all of the lines were non-branching, while for stem form (MSS) all of the accessions were cylindrical. For axil pigmentation (PPA), 79% were non-pigmented, while 21% of the lines were pigmented. With respect to stem striping (PRS), all of the accessions displayed this character. None of the lines displayed panicle branches (PB). For leaf shape (LS), all had triangular leaves. Similarly, all of the lines had indentations on the leaf margins (LM). None of the lines exhibited male sterility (MS). In terms of degree of dehiscence (DD), 75% of the accessions had normal dehiscence, 14% were strongly dehiscent, and 11% were slightly dehiscent. For panicle density (PD), 57% of the lines were lax, 36% intermediate, and 7% were compact. For panicle shape (PS), 71% were intermediate, 21% glomerulate, and 7% amaranthiform. With respect to perigonium pattern (PAP), 86% of the lines were semi-open and 14% closed. For insect susceptibility (PSY), 71% were lightly damaged, 25% moderately damaged, and 4% showed no insect damage. For pericarp appearance (PA), 93% of the lines were ashen and 7% granular. For episperm appearance (EA), 96% were opaque and 4% had vitreous episperm. For grain shape (GS), 54% were cylindrical, 42% elliptical, and 4% were lenticular. All of the lines displayed presence of saponin (SP). For saponin effusion (ES), 93% of the lines were medium, 4% produced minimal saponin, and the remaining 4% were rated as heavy saponin producers.

**Figure 2:**
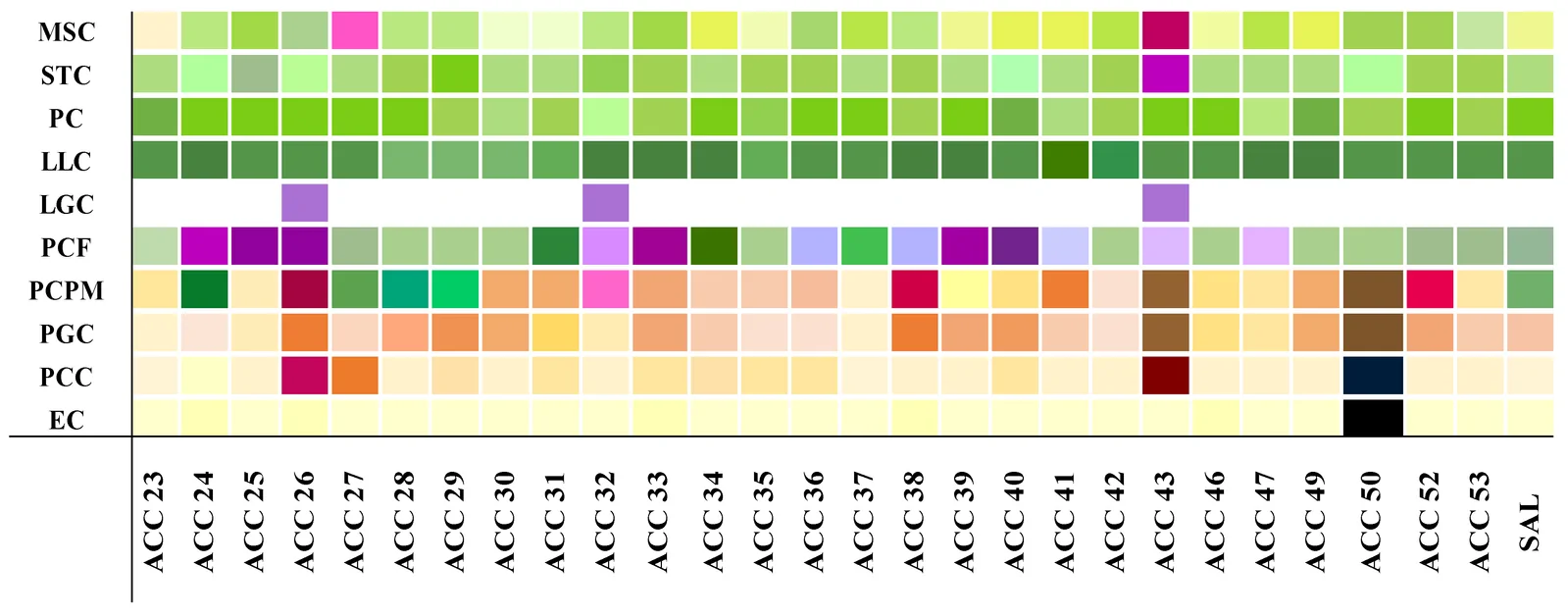
Polynomial plant color variables for the 27 accessions and one commercial variety based on the Royal Horticultural Society Color Chart Guide, Sixth Edition (2015; reprint 2019). Explanations: MSC = main stem color; STC = striae color; PC = petiole color; LLC = leaf lamina color; LGC = leaf granule color; PCF = panicle color at flowering; PCPM = panicle color at physiological maturity; PGC = perigonium color; PCC = pericarp color; EC = episperm color.

Plant color frequency analysis results are presented in Figure 2. Five accessions (18%) had intense yellow-green main stems (MSC), while four (14%) were brilliant yellow-green. For stem striae color (STC), 43% (12 accessions) were light yellow-green and 29% (8 accessions) were deep yellow-green. With respect to petiole color (PC), 46% (13 accessions) were intense yellow-green. Similarly, the laminar leaf color (LLC) of 46% (13 accessions) were medium olive green. Meanwhile, for leaf grain or epidermal bladder color (LGC) 89% (25 accessions) were colorless and 11% (3 accessions) were purple. With respect to panicle color at flowering (PCF), 29% (8 accessions) were predominantly medium yellow-green. At physiological maturity (PCPM), panicle color expression became highly variable, with three accessions (11%) appearing medium yellow-orange. For perigonium color (PGC), color was again highly variable, with 14% (4 accessions) being pale yellow and 11% (3 accessions) being medium orange-yellow. The pericarp color (PCC) in 12 accessions was mainly pale yellow (43%). The predominant episperm color (EC) was whitish-yellow at 79% (22 accessions).

### 2.6. Correlation Matrix

**Figure 3:**
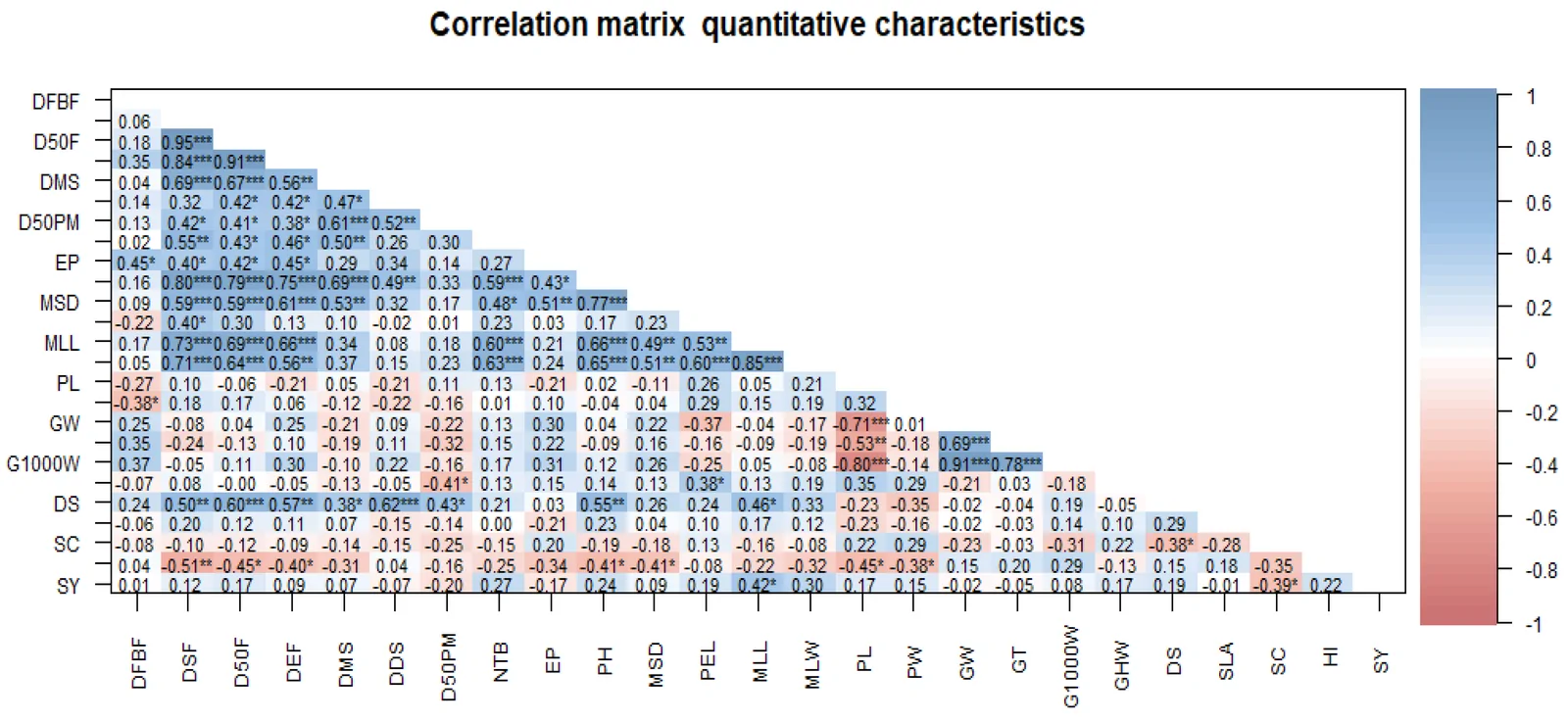
Matrix of correlation coefficients between quantitative variables for the 27 accessions and commercial variety SAL. Significance levels: * *p* < 0.05, ** *p* < 0.01, *** *p* < 0.001.

Spearman correlation coefficients are presented in Figure 3. Elevated correlation values were observed between discrete phenological stages such as D50F and DSF (r = 0.95), D50F and DEF (r = 0.91), DEF and DSF (r = 0.84); between morphological variables MLW and MLL (r = 0.85) and MSD and PH (r = 0.77); and between grain variables G1000W and GW (r = 0.91), G1000W and GT (r = 0.78), and between SY and MLL (0.42). Negative correlations were also observed, between G1000W and PL (r = −0.80), GW and PL (r = −0.78), and SY and SC (r = −0.39).

### 2.7. Analysis of Principal Components

**Figure 4:**
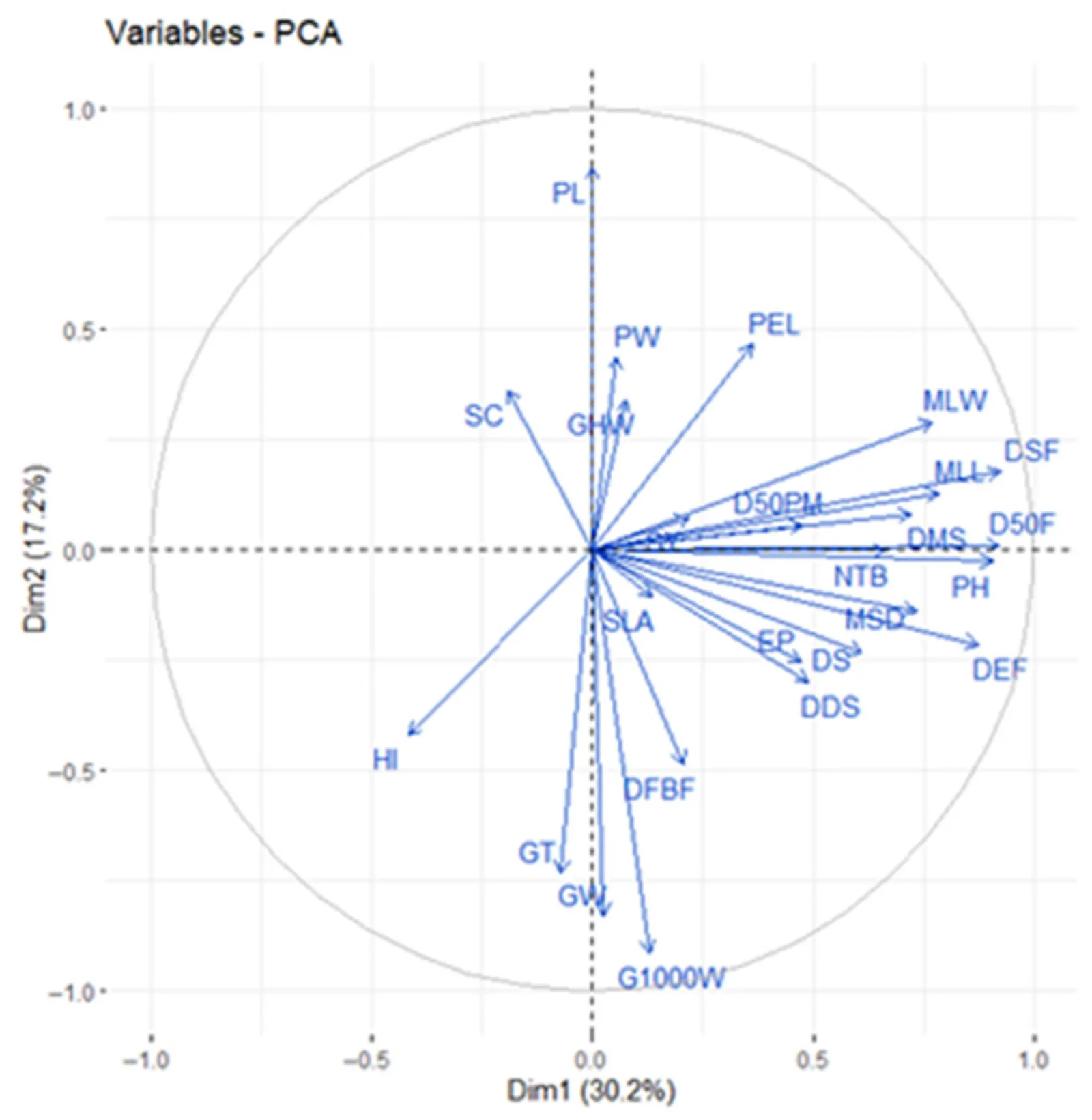
Analysis of principal components (PCA) between quantitative variables for the 27 accessions and commercial variety SAL.

The analysis of principal components is presented in Figure 4. The two principal components explained 47.4% of the total variance. Variables having the greatest contribution to the first principal component (PC1) were D50F, PH, DEF, DSF, MLL, DMS, DS and MLW. For PC2, variables PL, PW, HI, DFBF, GT, GW and G1000W were most significant.

**Figure 5:**
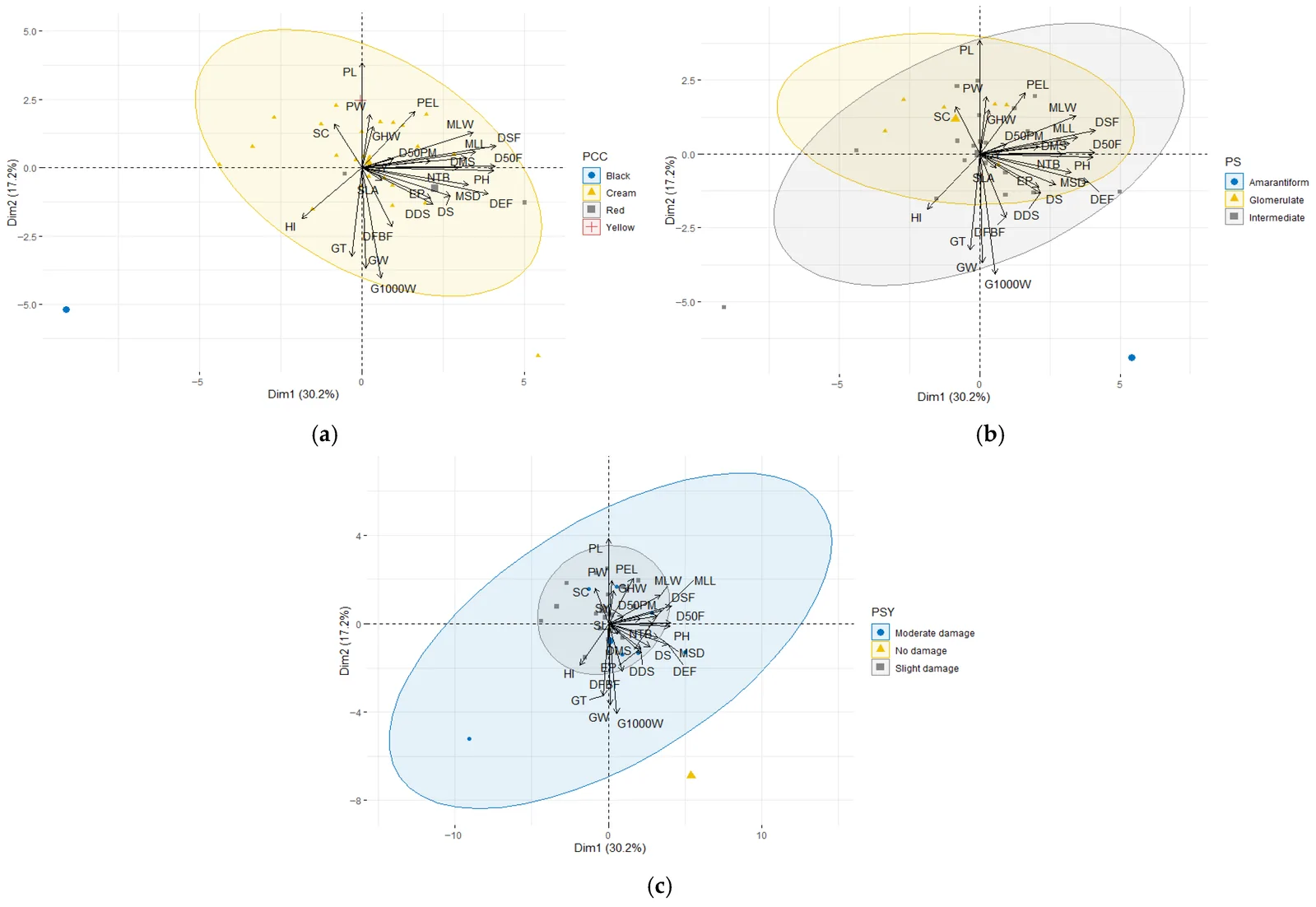
Principal component analyses (PCAs) for (**a**) grain color, (**b**) panicle shape, and (**c**) disease susceptibility.

Results of principal component analyses (PCAs) for qualitative morphological traits are presented in Figure 5. We observed modest discrimination among groups for grain color (Figure 5a), with an overwhelming number of lines having cream-colored seeds (Figure 2). Similarly, panicle morphology demonstrated only modest discrimination in the PCA biplot, with variables SC, PW, PL and GHW being associated with glomerulate panicles (Figure 5b). However, no discrimination was observed among groups for pest tolerance levels, as most genotypes were moderately pest-tolerant and a smaller group displayed slight pest damage and a general association with variables SC, PW, PEL, GHW and SY (Figure 5c).

### 2.8. Cluster Analysis

**Figure 6:**
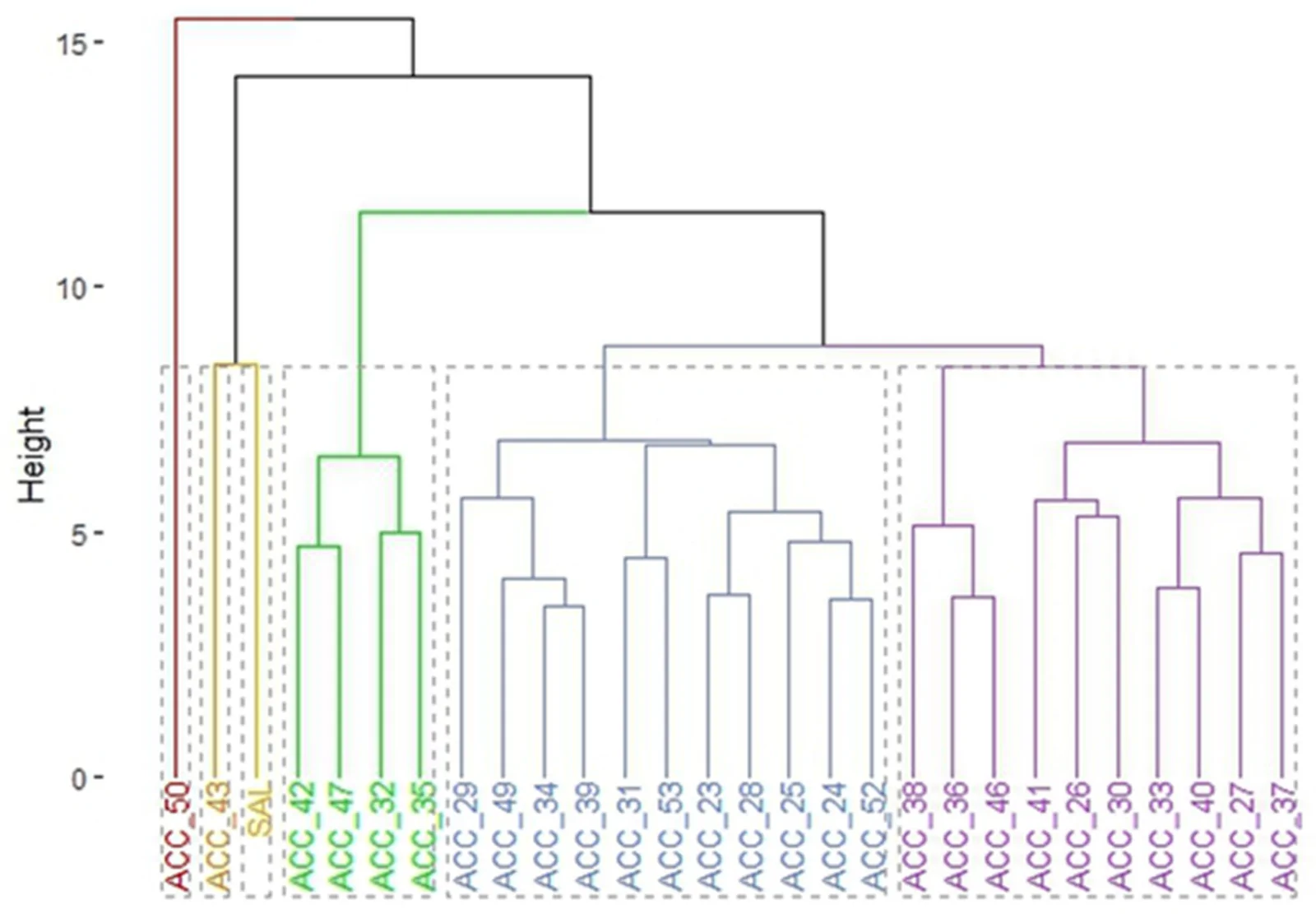
Dendrogram for the 27 accessions and one commercial variety, SAL. There are six discernable groups as depicted using the elbow method.

**Figure 7:**
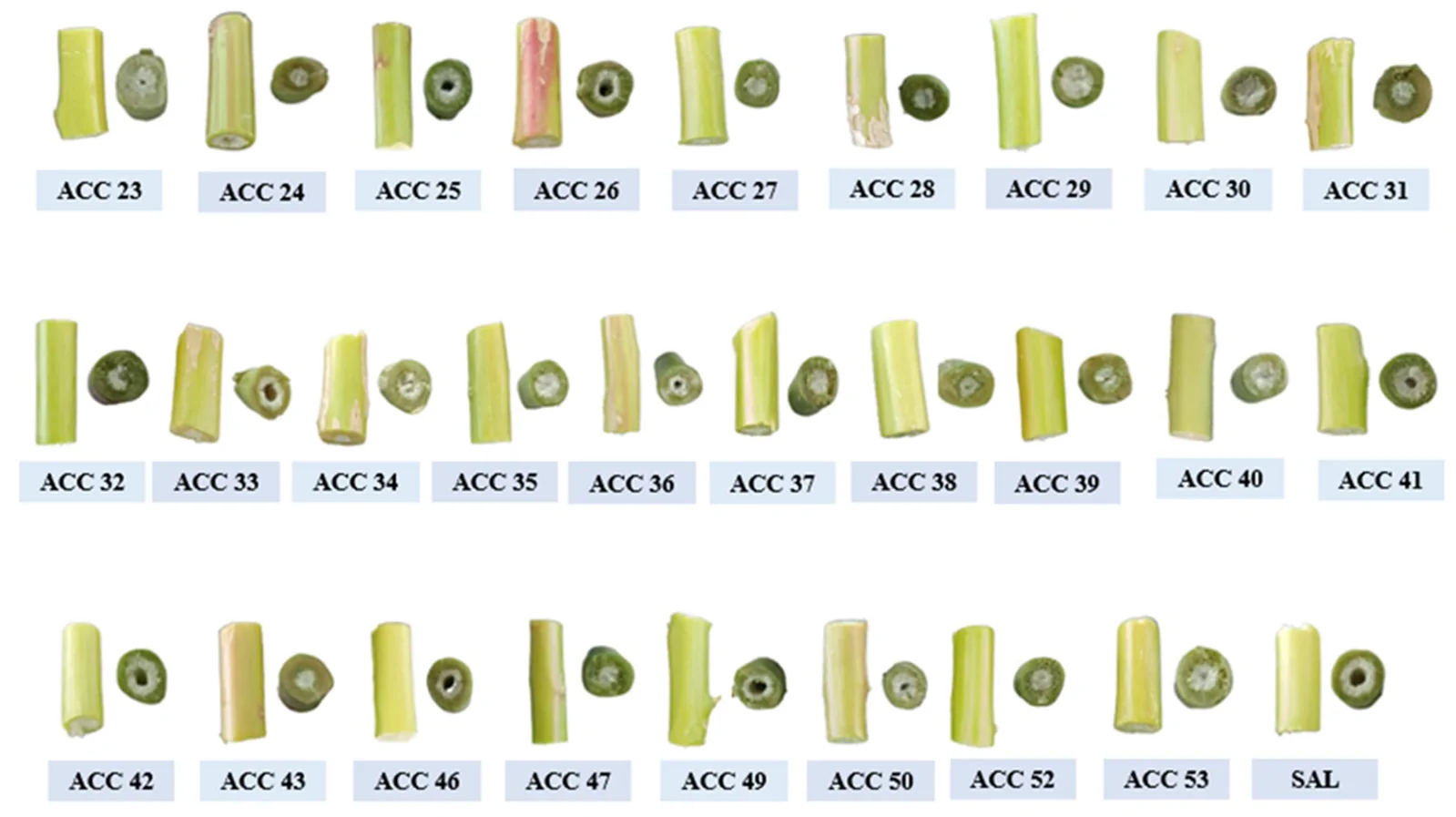
Main stem shape for the 27 accessions and commercial variety check, SAL.

**Figure 8:**
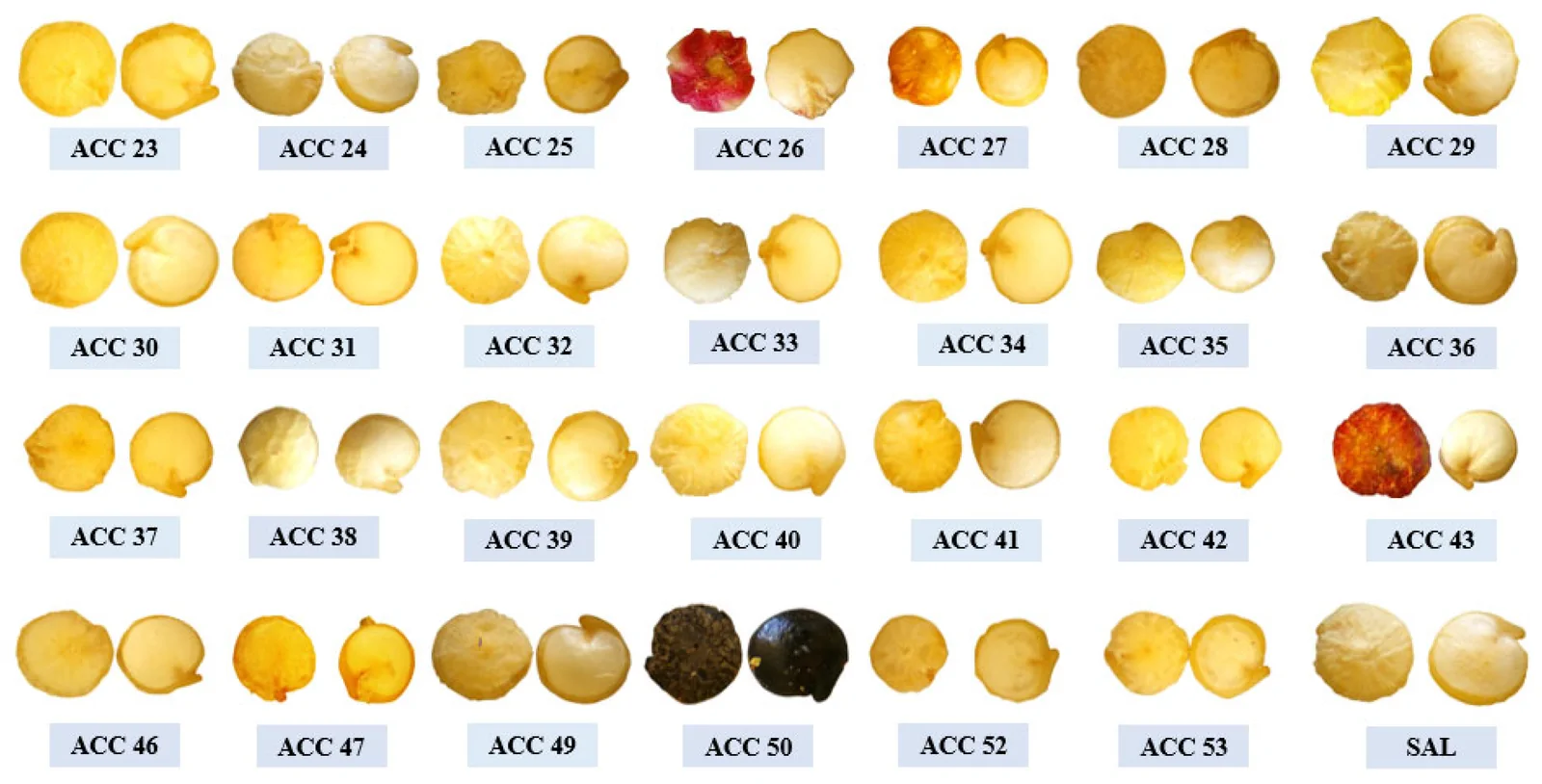
Image of the grain with pericarp (**left**) and without pericarp (**right**), for the 27 accessions and commercial variety check SAL. Seeds have been uniformly scaled.

**Figure 9:**
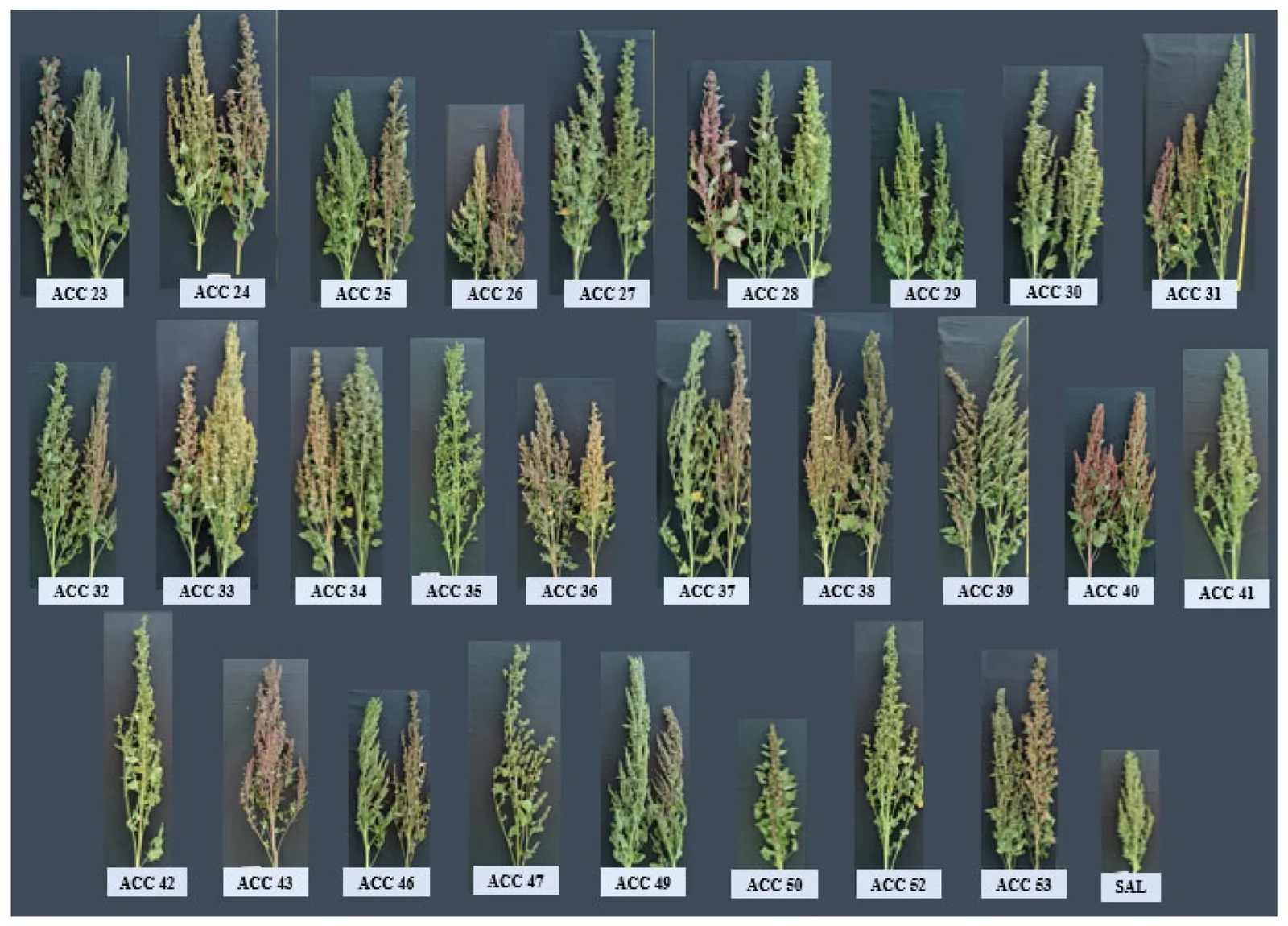
Observed phenotypic diversity in panicles of the twenty-eight quinoa lines at physiological maturity. Panicle photographs are set to a uniform size scale.

In the cluster analysis (Figure 6), six major groups were identified that shared similarities for many of the variables discussed above. Their phenotypic characteristics are shown photographically for stems (Figure 7), seeds (Figure 8), and panicles (Figure 9).

The first group (a) consisted of ACC 50. This line stands out for its earliness, having reached 50% physiological maturity by 42 DAS and for reaching complete maturity at 105 days (Table 1). Other salient characteristics of this accession/group are the following: shorter plant stature at 145.5 cm; narrower stem diameter of 10.65 mm; shorter leaf length at 5.76 cm; narrower leaves of 5.57 cm; shorter panicles of 50 cm; a narrower panicle diameter averaging 6.80 cm; reduced severity of mildew infection (1.22%); thicker grain (1.23 mm); and a higher harvest index (33.07%).

The second group (b) was composed of ACC 43 (Figure 6). Accession 43 was notable for its delayed maturation, requiring generally longer times to floral bud formation (57 DAS), to pasty grain stage (110 DAS), and to final physiological maturity (132 DAS, Table 1). Other important characteristics of this group included taller plant height at 231.1 cm, longer petioles at 5.97 cm, more severe mildew infestation (17.22%), and a harvest index of 28.77%.

The third group (c) consisted of the varietal check SAL. This variety was notable for its relatively high number of days until floral bud formation (37 DAS), to 50% physiological maturity (62 DAS), and for its long total growth cycle (132 days, Table 1). Additionally, SAL had the tallest plants (average 231.1 cm), the greatest average stem diameter (13.59 mm), and the largest (2.39 mm), thickest (1.18 mm), and heaviest seeds, as measured by thousand-seed (3.96 g) and hectoliter weights (0.62 g cm^−3^).

The fourth group (d) included four accessions: ACC 42, ACC 47, ACC 32 and ACC 35 (Figure 6). This group is characterized by reaching 50% physiological maturity between 48 and 52 DAS and with a 112-to-132-day requirement before reaching complete physiological maturity (Table 1). Stem diameter for this group varied from 10.62 to 12.15 mm, petiole length from 4.97 to 5.64 cm, maximum leaf lengths from 6.30 to 7.13 cm, average leaf widths from 6.27 to 7.36 cm, seed diameters from 1.92 to 2.09 mm, thousand-grain weights from 2.07 to 2.69 g, and hectoliter seed weight varied from 0.64 to 0.69 g cm^−3^ (Table 3). For this group, saponin percentage ranged from 0.123% to 0.180% and harvest index from 24.12% to 28.71%. In summary, these accessions were of medium height, had lower grain yields, were earlier, and they had smaller grains.

The fifth group (e) encompassed 11 accessions: ACC 29, ACC 49, ACC 34, ACC 39, ACC 31, ACC 53, ACC 23, ACC 28, ACC 25, ACC 24 and ACC 52 (Figure 6). This group required 51 to 56 days until initiation of the floral bud and 112 to 126 days until final physiological maturity (Table 1). Plant height in this group varied from 185.5 to 213.2 cm, stem diameters ranged from 11.83 to 13.49 mm, panicles were from 5.03 to 6.05 cm long, maximum leaf lengths ranged from 6.89 to 7.98 cm, maximum leaf widths from 6.89 to 7.98 cm, and hectoliter grain weight varied from 0.64 to 0.72 g cm^−3^. In terms of mildew susceptibility, these strains were highly variable (Table 2), with the most resistant lines being ACC 28 (1.56% severity) and ACC 23 (1.69% severity). Overall, this group consisted of average-height lines with intermediate yields, intermediate maturity, intermediate seed yields, and intermediate grain size.

The sixth group (f) of lines in the cluster analysis in Figure 6 was made up of the following ten accessions: ACC 38, ACC 36, ACC 46, ACC 41, ACC 26, ACC 30, ACC 33, ACC 40, ACC 27 and ACC 37. These lines flowered from 53 to 56 DAS and required 112 to 126 days to reach final physiological maturity. Their plant heights ranged from 187.1 to 215.5 cm, with the principal stem diameters measuring 11.26 to 12.75 mm. Petiole lengths in this group were from 4.50 to 6.09 cm, maximum leaf lengths from 6.99 to 8.32 cm, leaf widths from 6.39 to 8.13 cm, and panicle lengths ranged from 57.9 to 81.1 cm. For panicle diameters, the group varied between 9.47 and 12.80 cm. Seed characteristics were as follows: diameters ranged from 1.95 to 2.19 mm, widths from 0.93 to 1.08 mm, and the hectoliter weights varied from 0.62 to 0.71 g cm^−3^. In summary, this group was taller, had higher seed yields, was earlier maturing, and had smaller seeds.

### 2.9. Selection Indices

**Figure 10:**
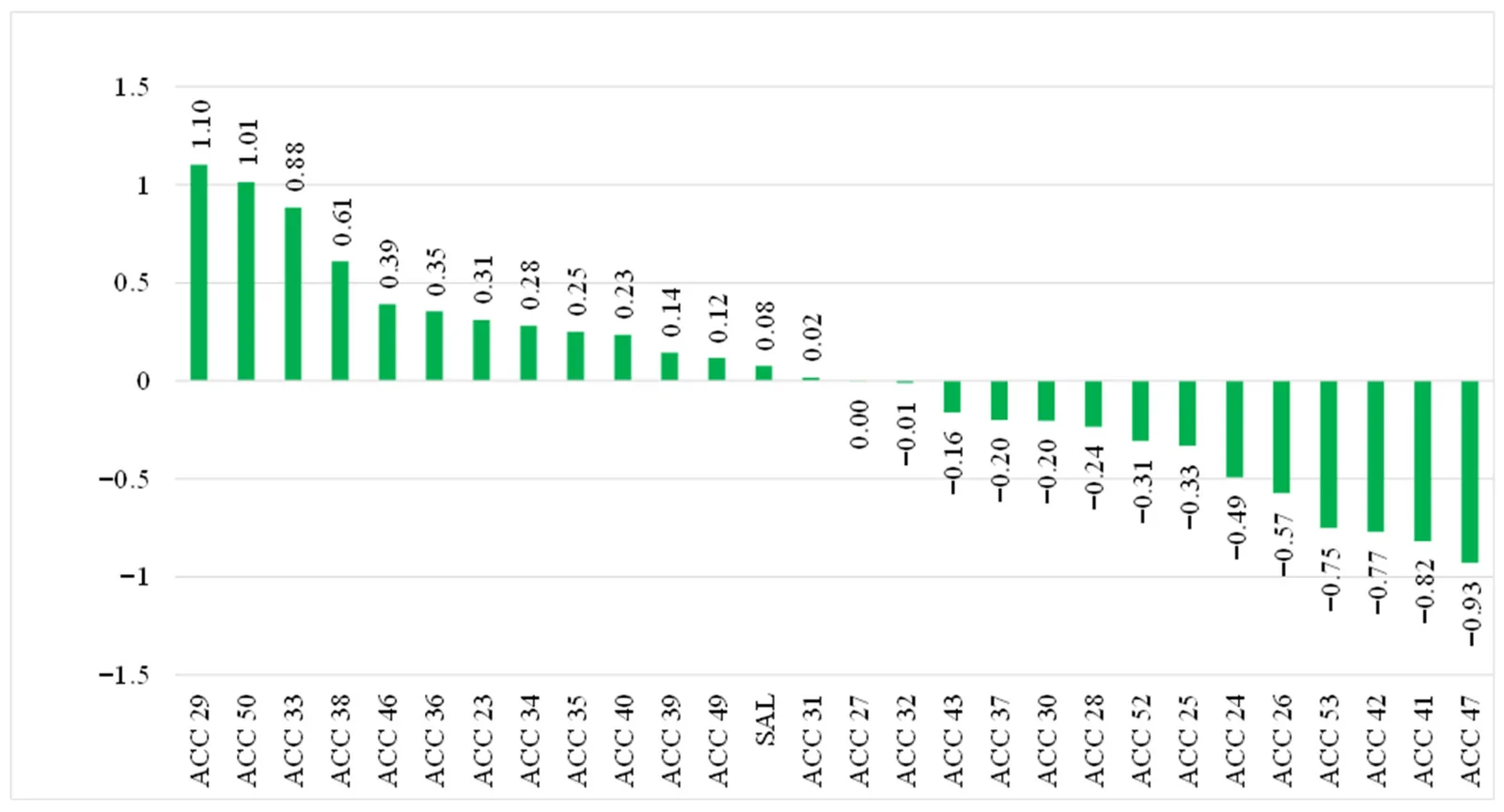
Selection Index (S.I.) for the 27 accessions and commercial check SAL.

Selection index values for the 27 accessions and the SAL check (Figure 10) ranged from between 1.10 to −0.93. Significant values are considered to be those equal to or greater than 0.60. Consequently, the four most attractive accessions (Figure 10) had plant heights of 145.5 to 222.1 cm, with an average of 188.3 cm. Their thousand-grain weights varied from 2.48 to 3.19 g, with an average of 2.93 g, and seed yields ranged from 3.20 to 5.60 t ha^−1^. The saponin contents varied between 0.101% and 0.117%, with an average value of 0.110%. In contrast, the five lines having the lowest SI values (−0.57 to −0.93) averaged 195.2 cm in height, with average thousand-seed weights of 2.41 g, average yields of 25.8 g plant^−1^, and saponin contents averaging 0.16% (Figure 10). Interestingly, varietal check SAL had only an average SI value of 0.08, being surpassed by 12 of the test accessions.

## 3. Discussion

The phenological quantitative variables of quinoa that were evaluated included plant emergence, which varied from 94 to 98% for the 27 accessions and was noted to be strongly affected by temperature [23]. Flower bud formation averaged 31 DAS, earlier than values observed by other authors but with similar genotypic variability [24]. Anthesis or flowering initiation occurred on average at 53 DAS, while previous studies reported the onset of anthesis at 100 DAS [25]. In our study, lines averaged 56 DAS until 50% of flowering, whereas others [26] had measured 84 DAS for the earliest and 94 DAS for the latest lines. For number of days from flowering to the pasty grain stage, our average was 92 days, while others have reported shorter periods [27], with 61 days in early varieties and 83 days in late varieties. We observed an average of 108 days from anthesis to 50% of physiological maturity, contrary to what was reported by [26], who obtained higher values. It is worth noting that others [28] had observed earlier maturation with average daily temperatures of 24 °C, which are similar to those in our experiment. When evaluating seedling emergence (EP), our values were very close to those of [29], who achieved 100% as a percentage of emergence for Colombian ecotypes. Of course, all phenological characteristics can be heavily influenced by the environment where the crop is grown and not solely by intrinsic genetics of the quinoa lines.

For the quantitative morphological variables in our quinoa trial, we noted an average plant height of 199 cm, which differs from values reported by others [30], who have reported a range of heights from 133.1 to 175.1 cm. Stem diameters in our study averaged 12.2 mm, which in another study had been reported to decrease by up to 17% under water-deficit conditions [31]. Panicle lengths in our study averaged 69 cm; this trait is known to be a function of the processes of growth and differentiation within the apical meristem [32]. For our 27 accessions, we identified a variability of 72%, indicative of differential genetic resistance/susceptibility among these lines, and contrasts with the results of others [33]. The average number of leaf margin indentations for our study was 26, whereas ref. [34] observed lower leaf teeth numbers in their study.

With respect to our quantitative seed measurements for quinoa, grain diameter across the 28 lines averaged 2.11 mm, which was higher than the report of [35]. Our average grain thickness was 1.07 mm, similar to what was recorded by [26], varying from 0.9 to 1 mm. For thousand-seed weight the average in our experiment was 2.72 g, results which are similar to those indicated by [36], with an average of 2.59 g, and within the range reported by [37], with values from 1 to 3 g, but which are lower than those of [38], who observed a range from 3.37 to 3.46 g. Our average hectoliter weight value was 0.67 kg L^−1^, which was close to the value of 0.73 kg L^−1^ from [39]. Saponin percentage in the 28 lines averaged 0.14%, which is higher than the 0.11% threshold for differentiating sweet from bitter lines [40], and only six of our 27 genotypes would be considered sweet. Our average harvest index was 26%, close to the value obtained by [41]. Our lines yielded an average of 3.7 t ha^−1^ of grain, a figure similar in magnitude to that recorded in trials in the Cochabamba Valley [26], where quinoa yielded 3.4–6.34 t ha^−1^. As with others [42] these data indicate that yield components are affected by various phenotypic characteristics, among them plant height, petiole length, leaf shape and size, etc. In addition, humidity can have significant effects on yield [43].

For qualitative morphological variables, all 27 lines exhibited a simple growth pattern, though there is evidence that this growth habit can vary when the soil is saline [44]. All our lines had cylindrical stems, as was also reported by [25] but not by [45], who observed 66% of lines with angular and 34% cylindrical stems. With respect to branching, none of our lines were highly branched, unlike ref. [46] who reported branched accessions in the lower and middle thirds of the stems. As had been reported by [45], all of our lines had triangular leaves. For panicle density, 57% were lax, 36% intermediate, and 7% compact, and these coincided with the observations of [45]. For panicle shape, 71% were of intermediate form, 21% glomerulate, and 7% were amaranthiform, in keeping with the results of [47], who reported intermediate (77%), glomerulate (15%), and amaranthiform (8%) panicles. However, in other studies, like [25], 76% amaranthiform and 24% glomerulate panicles are recorded, while ref. [45] reported 16% glomerulate, 38% intermediate, and 47% amaranthiform panicles in their studied genotypes. A majority of our lines had opaque seeds, in keeping with ref. [46] who reported 100% of their lines as being opaque. In terms of seed shape, 54% were cylindrical, 42% elliptical, and 4% were lenticular, which differed from [25], whose quinoas all had cylindrical seeds. All of our lines contained saponin as was previously noted by [25].

For color of the main stem, 18% of our lines were deep yellow-green and 14% were bright yellow-green; meanwhile, others [45] had reported green as the predominant stem color in in 31% of their genotypes. Striae color along the stem was mainly light yellow-green or strong yellow-green, results conforming with those of [25], who reported 76% of striae were green; however, others [48] have observed pink stem striae at maturity. For petiole color, 46% of our accessions were strong yellow-green, whereas ref. [46] had reported 81% of genotypes as having green petioles. For leaf blade color, 46% were medium olive green, as reported also by [49] but different from [50]. For glandular leaf trichome appearance, 89% were transparent while 11% were purple; others [45] reported 16% as being violet. For panicle color at flowering, 29% were medium yellow-green, a similar result to that obtained by [51], with green to violet panicles. Episperm color in 22 of our lines was whitish-yellow (cream), whereas ref. [52] had noted a range of distinct episperm colors, including translucent, white, cream, and including darker colors like black, gray, purple, and red, among others. This variation for episperm color implies that a wide range of genetic variability for seed pigmentation exists in quinoa [53].

We observed a strong positive correlation of 0.77 between main stem diameter and plant height. These results are concordant with [54], who obtained a moderately positive correlation (r = 0.56) between these two traits.

For the PCA, the first component encompassed 30.2% of the variability, explained primarily by the variables D50F, PH, DEF, DSF, MLL, DMS, DS, and MLW, whose vectors projected toward the positive pole of the axis and which comprise lines that were taller, with greater vegetative biomass, later maturity, and higher disease susceptibility. The second component of the PCA accounted for 17.2% of the variability, associated primarily with PL and PW, complementing the contributions of HI, DFBF, GT, GW, and G1000W. The extreme positive axis grouped lines have large panicles, while the negative axis contained lines showing higher productivity. Others [55] have reported the two principal components as explaining 51.1% of the total variation, with the first axis explaining 35.0% along a gradient of vegetative vigor and higher biomass, while the second accounted for 16.1% and projected a gradient for productivity and disease severity. These results contrast with those of [56], who observed that days to 50% flowering, seed yield per plant, and panicle length were the main contributing variables to PC1.

Our cluster analysis identified six main groups. The study of [57] had observed only three groups of clustered lines, whose groupings were highly influenced by panicle and seed characteristics along with plant height. Similarly, ref. [58] had identified three groups based on similar characteristics. However, ref. [59] had reported four groups dominated by genotypes from Peru and the Bolivian Altiplano (G1); the Bolivian Altiplano alone (G2); the USDA collection made by Ballón (and considered to be derived from crosses between Andean highland and coastal Chilean accessions, G3); and some accessions of Ballón along with some coastal Chilean lines (G4), with this last group being the most distinct genetically. Differences in these clustered groupings in the cited studies were most likely due to genetic differences of the source germplasm evaluated, along with different environmental conditions and other factors.

The selection indices of the 27 accessions and varietal check Salcedo-INIA varied between 1.10 and −0.93, with four accessions having the highest SI values (ACC 29, ACC 50, ACC33, and ACC 38) due to their combination of high yields, short statures, larger seeds, earliness, and lower saponin contents. In contrast, another study [59] treated the yield variables along with the number of panicles as positive SI contributors, but early maturation as a negative factor, in their study of 30 quinoa varieties.

## 4. Materials and Methods

### 4.1. Study Site

**Figure 11:**
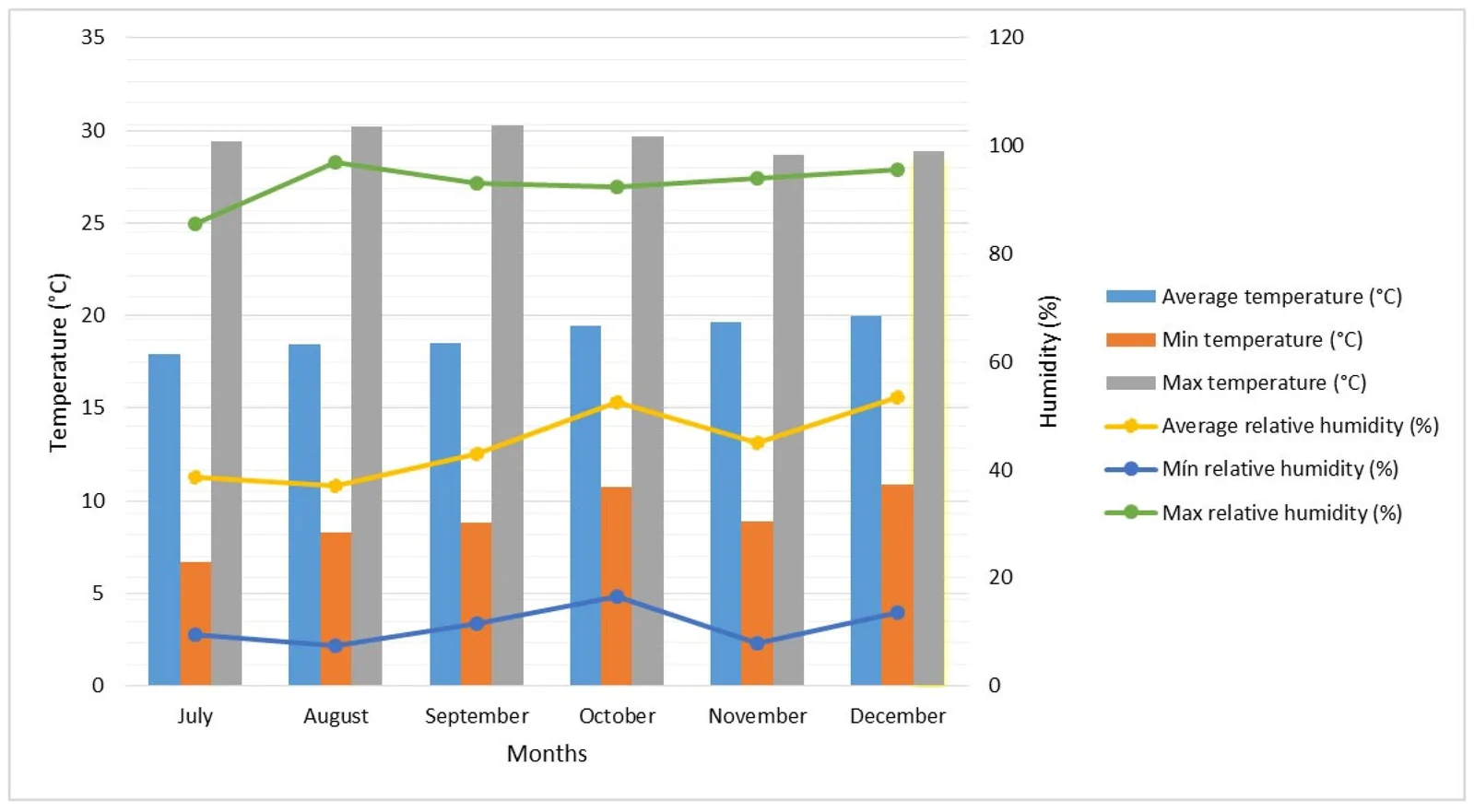
Data from the Pampa de Majes Automatic Meteorological Station in Majes, Arequipa, Peru.

The research trial was performed at the Center for Research, Teaching, and Agricultural Production “CIEPA-MAJES” of the National University of San Agustín of Arequipa, section B1 Specialized Zone; located in the Majes District, Caylloma Province, Arequipa Region, Peru, at 1431 m above sea level and 16°19′35″ south latitude and 72°13′01″ west longitude. The project was conducted under the specific agro-climatic conditions of this location (Table 5; Figure 11).

Water and soil analyses were performed in June, 2023 in the laboratory LABSAF-Arequipa, Santa Rita de Siguas. The soil was a sandy loam, with a moderately alkaline pH 7.7, non-saline electrical conductivity of 39.7 mS m^−1^, a low organic matter content of 1.1%, high in available phosphate at 12.1 mg kg^−1^, high in available potassium at 0.79 Cmol kg^−1^, a high calcium level of 10.42 Cmol kg^−1^, intermediate magnesium content of 2.05 Cmol kg^−1^, and with a modest cation exchange capacity of 14.28 meq 100 g^−1^. The irrigation water had a pH of 8, which is considered normal, along with a moderate electrical conductivity of 0.702 dS m^−1^, and moderately hard at 25.03 mg L^−1^.

Source: Automated meteorological station Pampa de Majes [60] and the meteorological station of Autodema at plot E3–67 [61].

### 4.2. Plant Material

The 27 selected experimental materials derived from native accessions were obtained from the Project Germplasm Bank of the National University of San Agustin of Arequipa (UNSA), using the commercial variety ‘Salcedo INIA’ (SAL) as a check (Table 6).

### 4.3. Variables Evaluated

Evaluations were done for 28 qualitative and 25 quantitative variables (Table 7) from 15 randomly selected plants, within the two central rows of each accession.

### 4.4. Agro-Morphological Characterization

Data on each of the quantitative and qualitative variables were scored and recorded based on quinoa descriptors for the crop published by [62]. Similarly, for the phenological stages were recorded the following: days to formation of the floral bud (DFBF); days to the start of flowering/anthesis (DSF); days to 50% flowering (D50F); days to the end of flowering (DEF); days to the milky grain stage (DMS); days to the pasty grain stage (DDS); days to 50% physiological maturity (D50PM); and, for each stage, days were counted from sowing. To determine hectoliter weight (GHW), a 10 mL precision test tube with an interior diameter of 12.18 mm was used for collected seed in a volume of 1 cm^3^, after which the sample was weighed on an analytical balance.

Colors of the main stem (MSC), striae (STC), petioles (PC), leaf blades (LLC), leaf granular trichomes (LGC), flowering panicles (PCF), plants at physiological maturity (PCPMs), perigonium (PGC), pericarp (PCC), and episperm (EC) were measured utilizing the Royal Horticultural Society color Chart, sixth edition (London, UK, 2015), with each color being numerically coded.

### 4.5. Evaluation of the Specific Leaf Area

The specific leaf area (SLA) was determined using the formula proposed by [63], for which 15 leaves from the middle third of the plant were collected, one from each plant sampled at 84 days post-sowing, which were later placed on a white surface and photographed from a height of 0.4 m. With the help of an IPAC, the photos were analyzed using Compu Eye, Leaf and Symptom Area (Doki, El Cairo, Egypt) in units of cm^2^ [64], after which the leaves were dried in a vacuum oven and then weighed on an analytical balance. The SLA was then calculated using the following formula:

SLA=LA/DW

where SLA = specific leaf area (cm^2^/g), LA = leaf area (cm^2^), and DW = dry weight (g).

### 4.6. Evaluation of Saponin Effusion (ES) and Saponin Content (SC)

Saponin effusion (ES) is the volume of foam measured using a modification of the protocol of [40]. Some 0.50 ± 0.02 g of whole seeds were measured out, after which they were crushed in a porcelain mortar to obtain flour. The quinoa flour was then placed in a test tube (15 cm tall × 13.77 mm interior diameter) to which was then added 5 mL of distilled water, the tube was then capped and agitated vigorously for 30 s, and then set aside to settle for 5 min. Afterwards the foam height was measured in cm. The data were then entered into the following equation to calculate saponin content (SC):

$$\%\;saponin\;=\;\frac{\left(0.441\;\times \;h\right)+\;0.001}{m\;\times \;10}$$

where h = foam height (cm) and m = sample weight (g).

### 4.7. Grain Yield (SY)

Yield per hectare was calculated based on weight of grain from 15 plants of the central row for each accession, taking into account the area occupied by each plant (0.09 m^2^). The data were then entered into the following formula [65]:

$$Yield\;({t\;ha}^{-1})\;=\;(\frac{Grain\;yield\;(\mathrm{kg})}{Area\;(m^{2})})\times 10$$

### 4.8. Mildew Severity (DS)

We evaluated mildew (*Peronospora variabilis*) severity according to the procedure described by [66], where a random leaf from each third of a plant was selected and the average percentage of damage on three leaves was calculated as the final value for infection severity per plant. This evaluation was done two weeks after sowing, in the absence of any fungicide application.

### 4.9. Pest Susceptibility (PSY)

For this evaluation we utilized the pest infection level scale according to Ponce and Badillo (2006), cited as [67], and this was performed two weeks after sowing, prior to any insecticide application.

### 4.10. Selection Index (SI)

The agromorphological characterization of the quinoa accessions would facilitate selection of genetic materials suitable for breeding and production in areas with unique environmental conditions [20]. Variables relating to yield, earliness, presence/absence of saponins, and other agronomic management variables (for example, plant height) were the most important for establishing a selection index (SI) based on linear equations proposed by [68]:

SI = yield (0.30)− plant height (0.15)+ 1000 seed weight (0.15)− days to 50% of physiological maturity (0.20)− presence of saponin (0.20)

where yield (t ha^−1^), height (cm) and saponin (%)

### 4.11. Agronomic Management of the Study

Each experimental unit consisted of a 5 × 3.6 m plot containing four rows that were 0.9 m wide, for a total area of 18 m^2^ per genotype.

Sowing was performed on 23 July 2023, at 0.01 m deep, at a density of 11 kg ha^−1^, with 20 g of seed per accession and 5 g per row. At 27 days after sowing (DAS), plots were thinned, leaving 4 to 5 plants per hill for a final density of approximately 555,000 plants ha^−1^. Each accession was separated by a row of hybrid maize, sown 30 days prior to the quinoa planting. Irrigation was performed using a DREAM v4. 109.1203 automated drip system controlled by the console software Talgil DREAM (version 4.0.6.8832) and the application SPOT (version 4.0.2136) for Android (Talgil Computing and Control Ltd. Naaman Center, Haifa—Acco Road, Israel). The water volume applied during crop development was 7785.4 m^3^ ha^−1^.

Fertilizers were applied at 301, 118, 360, 50, and 40 units per hectare of nitrogen, phosphorus, potassium, calcium, and magnesium, respectively and were based on the recommendations of the National Institute of Agrarian Innovation (INIA) of Peru with research done within the zone.

Phytosanitary control was performed using a manual hydraulic sprayer with an open cone head, taking into account rotation recommendations of action sites of the Action Committee Against Insect Resistance (IRAC) and the Action Committee Against Fungicide Resistance (FRAC). Prior to sowing, the quinoa seeds were impregnated with the fungicide Homai^®^ (Thiophanatemethyl + Thiram; Nippon Soda Co., Ltd., Tokyo, Japan), for preventative control of the rot fungus *Rhizoctonia solani*, at a dose of 0.01 g per 20 g of seeds. To control *Delia platura*, Orthene^®^ (Acephato; AMVAC Chemical Corporation, Newport Beach, CA, USA) was applied at 500 g ha^−1^. To prevent mildew (*Peronospora variabilis*), the fungicide Legasus^®^ (Metiram + Pyraclostrobin; BASF SE, Ludwigshafen, Germany) was applied at 27 DAS at the rate of 100 g per 20 L in a backpack sprayer; Helios^®^ (Propineb + Cymoxanil; Point Agro China Ltd., Wan Chai, China)) at 33 DAS at a dose of 100 g 20 L^−1^ of water; Acrobat^®^ (Dimethomorph + Mancozeb; BASF SE, Ludwigshafen, Alemania) at 44 DAS at a rate of 100 g 20 L^−1^ of water; and Curtine^®^ (Mancozeb + Cymoxanil; Zhejiang Well-Done Chemical Co., Ltd., Hangzhou, China) at 65 DAS at the rate of 50 g 20 L^−1^ of water.

Accessions were harvested manually, independently for each one and when the plants had reached physiological maturity. Cut panicles were allowed to dry in the sun, after which they were threshed and the seeds measured.

### 4.12. Data Analysis

To compare the qualitative variables, the mode was calculated and frequency analysis was performed, while for the quantitative data the medians ( $\bar{x}$ ), standard deviations (σ), and coefficients of variation (CVs) were calculated. Additionally, the quantitative variables were subjected to analyses of variance (ANOVA) and Duncan tests to compare the accessions (*p* ≤ 0.05), using R software version 4.4.1 and version 2024-06-14 ucrt of RStudio (Posit PBC, Boston, MA, USA). The packages used within this program included agricolae [69]; corrplot [70] was used to determine the correlation matrix; factoMineR [71] and factoextra [72] for the principal component analyses (PCA). Similarly, for the cluster dendrogram based on quantitative variables, the elbow method was used to determine the optimum number of groups using the factoextra package.

## 5. Conclusions

This study performed morphological and agronomic characterizations of 27 quinoa accessions within the hyper-arid Peruvian coastal region, so as to determine the capacity of these lines to respond or acclimatize to an adverse production environment in which they were neither originally domesticated nor selected through their hundreds of years of existence.

In terms of morphological characteristics, these lines matured at an average of 120 DAS, ranging from very early accessions like ACC 50 at 105 DAS and late ones like ACC 35, ACC 37, ACC 43, and SAL that matured by 132 DAS. We also identified lines with outstanding characteristics like ACC 29 and ACC 50, which stood out for their earliness, short stature, low saponin content, and superior productivity in comparison with the commercial check variety ‘Salcedo INIA’. On the other hand, all of the accessions performed similarly in their simple growth habit, cylindrical stems, triangular and toothed leaves, presence of striae, lack of male sterility, lack of branching, and presence of saponin. For the remaining characteristics the accessions displayed variability.

Based on analyses of the selection indices, we observed that ACC 29 and ACC 50 were superior to all the other tested lines and the Salcedo-INIA check, having SI values of 1.10 and 1.01, respectively.

Although this agro-morphological characterization was carried out in a single location, the results provide an essential catalog of the diversity present and identify those landrace accessions among the 27 that are most suitable for further line purification within the irrigated desert environment at Majes, Arequipa, Peru. By combining these most promising landrace selections together with internationally available germplasm of Chilean (coastal) origin and the extremely diverse lines being selected out of interspecific cross populations (quinoa X North American pitseed goosefoot, *C. berlandieri*), a highly diverse foundation is being laid for low-elevation quinoa breeding at the UNSA–Arequipa [36]. To supplement these findings and determine their adaptive attributes for other environments, these landrace accessions should be tested at different altitudes and latitudes.

## Figures and Tables

**Table 1:** Vegetative period in days for the evaluated accessions.

Phenological Stages (DAS)	Accessions																												Variability		
	23	24	25	26	27	28	29	30	31	32	33	34	35	36	37	38	39	40	41	42	43	46	47	49	50	52	53	SAL	$\bar{x}$	σ	CV (%)
Emergence	5	5	6	5	6	5	6	7	7	6	6	6	7	5	7	6	6	7	5	5	5	5	7	5	5	6	6	5	6	0.79	13.60%
Floral bud formation	30	30	30	30	30	30	30	30	30	30	30	33	30	30	30	33	33	30	30	33	30	33	33	33	32	33	30	37	31	1.79	5.73%
Start of flowering	54	52	52	53	53	56	51	54	54	48	53	52	48	55	55	54	54	54	54	52	57	56	52	53	42	53	54	56	53	2.97	5.62%
50% flowering	56	55	56	55	55	58	54	56	56	52	56	55	52	58	58	56	56	56	56	54	60	59	54	55	45	56	55	62	56	2.97	5.35%
End of flowering	59	59	63	58	59	63	57	62	61	55	59	61	54	62	61	61	62	61	63	56	65	64	59	62	51	61	59	70	60	3.68	6.10%
Milk stage	80	75	77	82	79	79	74	82	81	74	81	80	81	77	81	79	81	81	81	79	82	77	78	75	67	76	84	86	79	3.75	4.75%
Doughy grain	91	88	90	89	91	93	86	91	89	98	89	97	91	87	98	87	91	91	91	90	110	89	92	92	87	88	98	103	92	5.35	5.81%
50% physiological maturity	103	107	101	114	117	112	97	119	106	102	105	105	111	101	109	103	112	102	105	105	121	101	121	105	95	109	110	118	108	7.02	6.52%
End of physiological maturity	112	112	112	126	126	114	112	126	126	112	119	119	132	112	132	119	119	119	126	126	132	119	126	119	105	119	119	132	120	7.40	6.14%

**Table 2:** Morphological quantitative characters in 27 quinoa accessions and varietal check SAL. Arithmetic mean ± standard deviation. Explanations: PH = plant height (cm) at physiological maturity; MSD = main stem diameter (mm); PEL = petiole length (mm); MLL = mean leaf length (cm); MLW = mean leaf width (cm); PL = panicle length (cm); PW = panicle width (cm); DS = mildew disease severity (%); SLA = specific leaf area (cm^2^ g^−1^); NTB = number of leaf blade teeth.

Line	PH	MSD	PEL	MLL	MLW	PL	PW	DS	SLA	NTB
ACC 23	200.4 ± 12.7 efg	12.37 ± 1.59 abcde	5.69 ± 0.65 abc	6.89 ± 0.62 ijkl	7.49 ± 0.88 bcdefghi	70.7 ± 8.1 cdef	16.87 ± 2.10 b	1.69 ± 2.15 fgh	839 ± 113 abc	26 ± 5.9 efg
ACC 24	199.9 ± 24.5 efg	11.83 ± 2.40 cdef	5.45 ± 0.91 abcde	7.37 ± 0.71 efghi	7.63 ± 0.63 abcdefgh	81.9 ± 9.3 a	16.27 ± 2.46 b	2.31 ± 3.76 defgh	687 ± 135 defgh	26 ± 5.6 efg
ACC 25	185.5 ± 14.8 ijk	12.71 ± 1.99 abcd	6.05 ± 0.84 a	7.07 ± 0.61 ghij	7.01 ± 0.85 hijk	65.3 ± 5.1 fghi	15.53 ± 2.64 bc	4.53 ± 4.66 defgh	661 ± 93 efg	22 ± 6.2 hij
ACC 26	187.1 ± 10.7 hijk	12.07 ± 1.43 cde	5.99 ± 0.61 a	7.29 ± 0.83 efghi	7.32 ± 0.91 defghij	59.1 ± 6.2 ij	10.73 ± 1.67 fghij	2.04 ± 3.15 efgh	817 ± 139 abc	25 ± 6.1 fgh
ACC 27	195.5 ± 15.5 fghi	12.36 ± 2.41 abcde	5.55 ± 0.66 abcd	7.43 ± 0.74 defghi	7.68 ± 0.60 abcdef	76.8 ± 7.7 abc	11.80 ± 2.14 defgh	3.98 ± 5.00 defgh	721 ± 97 cdefg	24 ± 6.1 fghi
ACC 28	194.2 ± 14.9 ghij	12.66 ± 1.21 abcd	5.83 ± 0.53 a	7.64 ± 0.63 cdefg	7.73 ± 0.63 abcdef	69.5 ± 8.7 cdefg	18.77 ± 1.84 a	1.56 ± 1.81 gh	671 ± 134 efg	28 ± 6.1 def
ACC 29	193.5 ± 17.3 ghij	12.88 ± 1.57 abcd	5.52 ± 0.75 abcd	7.16 ± 0.78 fghij	7.03 ± 0.81 ghijk	62.5 ± 10.4 ghij	12.07 ± 1.03 defgh	2.53 ± 4.20 defgh	664 ± 95 efg	24 ± 5.9 fgh
ACC 30	198.9 ± 14.4 efgh	11.78 ± 1.33 cdef	5.11 ± 0.54 bcdef	6.99 ± 0.66 hijk	6.39 ± 0.66 l	69.3 ± 8.2 cdefg	12.27 ± 1.83 defgh	6.89 ± 8.11 cdef	879 ± 161 ab	28 ± 5.0 def
ACC 31	199.3 ± 13.2 efgh	12.89 ± 2.04 abcd	5.99 ± 0.91 a	7.81 ± 0.85 abcde	8.01 ± 1.09 ab	75.2 ± 7.4 abcd	11.27 ± 1.44 efgh	3.51 ± 3.78 defgh	679 ± 116 efg	32 ± 5.2 abc
ACC 32	179.7 ± 19.1 k	10.76 ± 1.21 f	4.97 ± 0.72 def	6.45 ± 0.45 klm	6.27 ± 0.46 l	66.6 ± 7.5 efgh	14.73 ± 2.66 c	2.47 ± 2.11 defgh	648 ± 153 efg	20 ± 5.7 jk
ACC 33	192.0 ± 14.1 ghij	11.26 ± 1.20 ef	5.48 ± 0.71 abcd	7.55 ± 0.65 defgh	7.85 ± 0.51 abcde	78.7 ± 11.3 ab	11.87 ± 1.77 defgh	5.93 ± 7.80 cdefgh	805 ± 176 abcd	31 ± 5.6 abcd
ACC 34	200.5 ± 15.2 efg	12.11 ± 1.27 cde	5.67 ± 1.00 abc	7.07 ± 0.80 ghij	7.15 ± 0.81 fghijk	71.0 ± 12.0 cdef	11.07 ± 1.87 fghi	4.82 ± 7.01 defgh	696 ± 82 defg	28 ± 5.4 cdef
ACC 35	186.9 ± 15.8 hijk	12.15 ± 1.98 cde	5.14 ± 0.70 bcde	6.38 ± 0.91 lm	6.34 ± 0.92 l	73.8 ± 6.2 bcde	10.67 ± 2.09 fghij	3.89 ± 3.65 defgh	635 ± 151 fg	20 ± 5.6 ijk
ACC 36	202.1 ± 12.2 defg	11.69 ± 1.40 def	5.82 ± 0.60 a	7.31 ± 0.66 efghi	7.91 ± 0.60 abcd	61.5 ± 6.9 hij	12.33 ± 1.59 def	6.29 ± 6.56 cdefgh	923 ± 119 a	22 ± 5.1 ghij
ACC 37	204.3 ± 13.0 cdefg	12.07 ± 1.46 cde	5.99 ± 0.63 a	7.18 ± 0.59 fghij	7.28 ± 0.61 defghij	78.7 ± 11.3 ab	11.67 ± 2.66 defgh	7.09 ± 10.20 bcde	599 ± 108 g	22 ± 5.0 ghij
ACC 38	222.1 ± 16.3 ab	12.75 ± 1.09 abcd	6.09 ± 0.84 a	8.32 ± 0.77 a	8.13 ± 0.57 ab	81.1 ± 7.9 ab	12.80 ± 2.21 de	6.67 ± 8.67 cdefg	888 ± 233 a	25 ± 5.2 fgh
ACC 39	208.5 ± 9.9 cde	12.23 ± 1.05 bcde	5.03 ± 0.76 cdef	7.25 ± 0.52 efghij	6.99 ± 0.60 ijk	80.0 ± 10.0 ab	11.20 ± 1.26 efgh	2.27 ± 2.94 efgh	610 ± 129 g	32 ± 5.2 ab
ACC 40	207.5 ± 14.2 cdef	12.10 ± 1.55 cde	5.55 ± 1.12 abcd	8.15 ± 0.73 abc	7.81 ± 0.77 abcde	59.1 ± 7.2 ij	10.47 ± 2.00 hijk	4.38 ± 6.11 defgh	751 ± 143 cdef	30 ± 5.0 bcde
ACC 41	215.5 ± 13.3 bc	12.74 ± 1.25 abcd	4.50 ± 0.54 f	6.68 ± 0.69 jklm	6.81 ± 0.51 jkl	70.5 ± 4.3 cdef	9.47 ± 2.88 ijkl	2.20 ± 2.52 efgh	837 ± 178 abc	22 ± 4.7 ghij
ACC 42	182.3 ± 14.7 jk	11.70 ± 1.00 def	5.45 ± 0.75 abcde	6.30 ± 0.56 m	6.58 ± 0.53 kl	80.9 ± 9.9 ab	9.07 ± 2.09 kl	2.53 ± 2.84 defgh	758 ± 152 bcdef	19 ± 4.5 jk
ACC 43	231.1 ± 15.2 a	13.08 ± 1.26 abc	5.97 ± 0.81 a	7.80 ± 0.74 abcde	8.13 ± 0.89 ab	67.7 ± 6.7 defgh	8.80 ± 1.15 l	17.22 ± 10.80 a	822 ± 190 abc	30 ± 4.7 bcde
ACC 46	209.1 ± 16.1 cde	12.19 ± 1.19 bcde	5.76 ± 0.70 ab	8.26 ± 0.90 ab	7.24 ± 0.75 efghij	57.9 ± 14.3 j	10.60 ± 1.72 ghijk	11.64 ± 7.86 b	913 ± 120 a	24 ± 4.9 fgh
ACC 47	177.8 ± 13.5 k	10.62 ± 0.98 f	5.64 ± 1.14 abc	7.13 ± 0.76 fghij	7.36 ± 0.81 cdefghij	73.5 ± 17.8 bcde	8.60 ± 1.30 l	7.56 ± 8.57 bcd	640 ± 154 efg	24 ± 4.9 fgh
ACC 49	203.9 ± 16.4 cdefg	12.63 ± 1.83 abcd	5.45 ± 0.82 abcde	7.73 ± 0.97 bcdef	8.10 ± 1.12 ab	70.7 ± 6.7 cdef	10.87 ± 1.77 fghi	3.20 ± 4.32 defgh	657 ± 98 efg	32 ± 4.8 ab
ACC 50	145.5 ± 9.4 l	10.65 ± 0.72 f	4.91 ± 0.78 def	5.76 ± 0.55 n	5.57 ± 0.50 m	50.0 ± 5.6 k	6.80 ± 0.77 m	1.22 ± 1.19 h	821 ± 112 abc	18 ± 4.2 k
ACC 52	200.1 ± 18.4 efg	12.33 ± 1.12 abcde	5.83 ± 0.76 a	7.98 ± 0.44 abcd	8.23 ± 0.78 a	80.3 ± 8.1 ab	13.27 ± 2.94 d	4.09 ± 4.72 defgh	762 ± 225 bcde	24 ± 4.2 fgh
ACC 53	213.2 ± 14.0 bcd	13.49 ± 1.86 ab	5.88 ± 0.61 a	7.40 ± 0.55 defghi	7.97 ± 0.80 abc	70.0 ± 10.2 cdef	9.20 ± 1.01 jkl	5.91 ± 6.62 cdefgh	839 ± 130 abc	35 ± 4.2 a
SAL	231.1 ± 10.9 a	13.59 ± 1.35 a	4.82 ± 0.70 ef	7.71 ± 0.79 bcdef	7.65 ± 0.86 abcdefg	38.9 ± 5.9 l	8.47 ± 1.06 l	10.69 ± 6.86 bc	715 ± 161 cdefg	27 ± 4.2 def
$\bar{x}$	198.84	12.20	5.54	7.29	7.35	69.32	11.70	4.97	747.73	25.74
σ	17.15	0.75	0.42	0.61	0.67	10.09	2.74	3.56	97.36	4.37
CV	9%	6%	8%	8%	9%	15%	23%	72%	13%	17%

Values followed by the same letter were not significantly different, based on the Duncan Test (*p* ≤ 0.05).

**Table 3:** Quantitative yield characters in the 27 quinoa accessions and cultivar check SAL. Arithmetic mean ± standard deviation. Explanations: GW = grain diameter (mm); GT = grain thickness (mm); G1000W = thousand-seed weight (g); GHW = grain hectoliter weight (g cm^−3^); SC = saponin percentage (%); HI = harvest index (%); SY = seed yield (t ha^−1^).

Line	GW	GT	G1000W	GHW	SC	HI	SY
ACC 23	2.21 ± 0.07 de	1.05 ± 0.06 ijk	2.85 ± 0.28 ef	0.69 ± 0.04 bcd	0.133 ± 0.046 defghi	26.53 ± 1.59 bcdef	4.21 ± 0.97 bcdefg
ACC 24	2.13 ± 0.11 fghi	1.03 ± 0.05 kl	2.57 ± 0.35 fghi	0.68 ± 0.03 cdef	0.178 ± 0.066 bc	21.90 ± 2.85 hi	3.49 ± 1.26 cdefghi
ACC 25	2.13 ± 0.07 fghi	1.13 ± 0.04 c	2.77 ± 0.32 efg	0.67 ± 0.02 defghij	0.210 ± 0.057 a	24.58 ± 1.69 fg	3.54 ± 0.73 cdefghi
ACC 26	2.06 ± 0.07 jkl	1.08 ± 0.05 defghi	2.84 ± 0.22 ef	0.62 ± 0.05 n	0.165 ± 0.046 bcd	27.59 ± 2.37 bcde	3.02 ± 1.05 fghi
ACC 27	1.96 ± 0.09 nop	0.93 ± 0.06 n	2.10 ± 0.40 jkl	0.64 ± 0.03 ijklmn	0.097 ± 0.022 jk	26.15 ± 1.74 def	4.40 ± 1.92 abcdef
ACC 28	2.18 ± 0.06 efg	1.09 ± 0.04 cdefgh	2.75 ± 0.30 efg	0.68 ± 0.04 cdefg	0.130 ± 0.057 defghij	23.12 ± 2.72 ghi	3.37 ± 1.02 defghi
ACC 29	2.29 ± 0.07 bc	1.10 ± 0.05 cde	3.19 ± 0.32 c	0.66 ± 0.04 fghijkl	0.101 ± 0.031 ijk	24.37 ± 2.50 fg	4.77 ± 1.58 abc
ACC 30	2.19 ± 0.06 efg	1.06 ± 0.06 ghijk	2.90 ± 0.33 de	0.62 ± 0.04 mn	0.108 ± 0.019 ghijk	24.00 ± 1.85 fghi	3.53 ± 1.01 cdefghi
ACC 31	2.07 ± 0.06 ijkl	1.10 ± 0.05 cdefg	2.57 ± 0.43 fghi	0.72 ± 0.03 a	0.136 ± 0.031 defgh	25.02 ± 3.04 fg	3.97 ± 1.25 cdefgh
ACC 32	2.09 ± 0.11 ijk	1.06 ± 0.05 hijk	2.69 ± 0.55 efgh	0.68 ± 0.04 cdef	0.180 ± 0.059 b	27.59 ± 2.81 bcde	3.86 ± 1.03 cdefgh
ACC 33	1.99 ± 0.08 mno	1.08 ± 0.05 defghi	2.58 ± 0.44 fghi	0.65 ± 0.03 hijkl	0.114 ± 0.033 fghijk	26.40 ± 3.57 bcdef	5.60 ± 2.23 a
ACC 34	2.10 ± 0.09 hij	1.11 ± 0.05 cde	2.84 ± 0.36 ef	0.70 ± 0.02 abc	0.164 ± 0.039 bcd	25.76 ± 2.86 ef	4.87 ± 2.05 abc
ACC 35	2.02 ± 0.10 lmn	1.00 ± 0.04 lm	2.35 ± 0.38 ijk	0.64 ± 0.04 lmn	0.123 ± 0.019 efghij	28.71 ± 1.88 bc	4.65 ± 2.78 abcd
ACC 36	2.13 ± 0.06 ghi	1.03 ± 0.03 kL	2.84 ± 0.32 ef	0.68 ± 0.04 cdefgh	0.138 ± 0.033 defg	26.30 ± 2.24 cdef	4.31 ± 1.61 abcdefg
ACC 37	2.03 ± 0.07 klm	1.05 ± 0.04 hijk	2.36 ± 0.42 ij	0.65 ± 0.03 hijklm	0.148 ± 0.038 bcdef	23.12 ± 4.51 ghi	4.16 ± 2.01 cdefg
ACC 38	1.95 ± 0.08 op	1.04 ± 0.03 ijk	2.49 ± 0.31 ghi	0.71 ± 0.04 ab	0.117 ± 0.023 fghijk	26.42 ± 2.87 bcdef	5.52 ± 2.00 ab
ACC 39	2.19 ± 0.04 ef	1.12 ± 0.03 cd	2.79 ± 0.28 ef	0.67 ± 0.03 defghi	0.140 ± 0.038 defg	24.90 ± 2.81 fg	4.80 ± 1.55 abc
ACC 40	2.16 ± 0.09 efgh	1.06 ± 0.07 fghijk	2.69 ± 0.35 efgh	0.64 ± 0.02 klmn	0.144 ± 0.046 cdef	28.56 ± 4.00 bcd	4.45 ± 2.02 abcde
ACC 41	2.09 ± 0.05 ijk	1.03 ± 0.03 jkl	2.37 ± 0.17 ij	0.65 ± 0.05 ghijkl	0.159 ± 0.038 bcd	21.66 ± 1.90 i	2.67 ± 0.87 hi
ACC 42	1.92 ± 0.10 p	1.04 ± 0.05 ijk	2.09 ± 0.52 kl	0.69 ± 0.03 bcde	0.164 ± 0.034 bcd	24.12 ± 2.87 fgh	2.44 ± 1.33 i
ACC 43	2.10 ± 0.06 hij	1.07 ± 0.03 efghij	2.93 ± 0.42 cde	0.67 ± 0.03 defghijk	0.089 ± 0.025 k	28.77 ± 2.05 b	4.11 ± 1.42 cdefg
ACC 46	2.17 ± 0.05 efg	1.06 ± 0.06 ghijk	2.97 ± 0.47 cde	0.69 ± 0.03 bcd	0.135 ± 0.040 defghi	26.89 ± 1.81 fg	4.38 ± 2.22 abcdef
ACC 47	1.95 ± 0.08 op	0.98 ± 0.05 m	2.07 ± 0.21 l	0.64 ± 0.05 ijklmn	0.156 ± 0.040 bcde	26.37 ± 2.24 bcdef	2.99 ± 1.41 ghi
ACC 49	2.26 ± 0.06 cd	1.10 ± 0.06 cdef	3.14 ± 0.24 cd	0.64 ± 0.03 jklmn	0.155 ± 0.072 bcde	25.24 ± 3.10 efg	4.05 ± 1.31 cdefg
ACC 50	2.33 ± 0.05 b	1.23 ± 0.03 a	3.48 ± 0.23 b	0.66 ± 0.04 efghijkl	0.108 ± 0.017 ghijk	33.07 ± 0.96 a	3.20 ± 0.74 efghi
ACC 52	2.04 ± 0.06 jklm	1.08 ± 0.05 defghi	2.43 ± 0.27 hi	0.65 ± 0.03 hijkl	0.157 ± 0.027 bcde	22.90 ± 2.29 ghi	3.88 ± 1.51 cdefgh
ACC 53	2.06 ± 0.06 jkl	1.09 ± 0.03 cdefgh	2.67 ± 0.27 efgh	0.70 ± 0.03 abcd	0.155 ± 0.039 bcde	22.87 ± 2.06 ghi	3.20 ± 1.07 efghi
SAL	2.39 ± 0.13 a	1.18 ± 0.03 b	3.96 ± 0.34 a	0.62 ± 0.03 n	0.103 ± 0.015 hijk	25.33 ± 1.55 efg	3.77 ± 1.16 cdefghi
$\bar{x}$	2.11	1.07	2.72	0.67	0.14	25.65	3.71
σ	0.12	0.06	0.41	0.03	0.03	2.45	0.26
CV	6%	5%	15%	4%	21%	10%	7%

Values followed by the same letter were not significantly different, based on the Duncan Test (*p* ≤ 0.05).

**Table 4:** Qualitative morphological and grain variables for the 27 quinoa accessions and varietal check SAL. Explanations: PPA = axil pigmentation (+ = present, − = absent); DD = degree of dehiscence (+++ = strong, ++ = regular, + = slight); PD = panicle density; PS = panicle shape; PAP = perigonium appearance; PSY = insect damage; PA = pericarp appearance; EA = episperm appearance; GS = grain shape; ES = saponin effusion.

Line	PPA	DD	PD	PS	PAP	PSY	PA	EA	GS	ES
ACC 23	+	+++	Lax	Intermediate	Semi-open	Slight	Ashen	Opaque	Cylindrical	Intermediate
ACC 24	+	++	Lax	Intermediate	Semi-open	Slight	Ashen	Opaque	Cylindrical	Intermediate
ACC 25	+	+++	Lax	Intermediate	Semi-open	Slight	Ashen	Opaque	Ellipsoid	Intermediate
ACC 26	+	++	Lax	Intermediate	Semi-open	Slight	Ashen	Opaque	Ellipsoid	High
ACC 27	−	++	Intermediate	Intermediate	Semi-open	Slight	Grainy	Opaque	Cylindrical	Intermediate
ACC 28	−	+++	Intermediate	Intermediate	Closed	Slight	Grainy	Opaque	Cylindrical	Intermediate
ACC 29	−	++	Intermediate	Intermediate	Semi-open	Slight	Ashen	Opaque	Ellipsoid	Intermediate
ACC 30	+	++	Intermediate	Intermediate	Semi-open	Slight	Ashen	Opaque	Cylindrical	Intermediate
ACC 31	−	++	Compact	Intermediate	Semi-open	Slight	Ashen	Opaque	Cylindrical	Intermediate
ACC 32	−	++	Lax	Intermediate	Semi-open	Slight	Ashen	Opaque	Cylindrical	Intermediate
ACC 33	−	++	Intermediate	Intermediate	Semi-open	Slight	Ashen	Opaque	Cylindrical	Intermediate
ACC 34	−	++	Lax	Intermediate	Semi-open	Slight	Ashen	Opaque	Cylindrical	Intermediate
ACC 35	−	++	Intermediate	Intermediate	Semi-open	Slight	Ashen	Opaque	Cylindrical	Intermediate
ACC 36	−	+	Lax	Glomerulate	Semi-open	Slight	Ashen	Opaque	Ellipsoid	Intermediate
ACC 37	−	+	Intermediate	Intermediate	Semi-open	Slight	Ashen	Opaque	Lenticular	Intermediate
ACC 38	+	++	Intermediate	Intermediate	Semi-open	Slight	Ashen	Opaque	Ellipsoid	Low
ACC 39	+	++	Lax	Intermediate	Semi-open	Slight	Ashen	Vitreous	Ellipsoid	Intermediate
ACC 40	−	++	Lax	Intermediate	Semi-open	Slight	Ashen	Opaque	Cylindrical	Intermediate
ACC 41	−	++	Lax	Glomerulate	Semi-open	Slight	Ashen	Opaque	Ellipsoid	Intermediate
ACC 42	−	+++	Compact	Amarathiform	Semi-open	Slight	Ashen	Opaque	Ellipsoid	Intermediate
ACC 43	−	++	Lax	Intermediate	Closed	Moderate	Ashen	Opaque	Cylindrical	Intermediate
ACC 46	+	++	Lax	Intermediate	Closed	Moderate	Ashen	Opaque	Ellipsoid	Intermediate
ACC 47	−	++	Lax	Glomerulate	Semi-open	Moderate	Ashen	Opaque	Cylindrical	Intermediate
ACC 49	−	++	Lax	Glomerulate	Semi-open	Moderate	Ashen	Opaque	Ellipsoid	Intermediate
ACC 50	−	++	Lax	Glomerulate	Closed	Moderate	Ashen	Opaque	Ellipsoid	Intermediate
ACC 52	+	++	Intermediate	Glomerulate	Semi-open	Moderate	Ashen	Opaque	Cylindrical	Intermediate
ACC 53	−	++	Lax	Intermediate	Semi-open	Moderate	Ashen	Opaque	Cylindrical	Intermediate
SAL	−	+	Intermediate	Amaranthiform	Semi-open	None	Ashen	Opaque	Ellipsoid	Intermediate

**Table 5:** Meteorological data for Majes District during the 2023 study period.

	July	August	September	October	November	December
Average temperature (°C)	17.94	18.44	18.49	19.45	19.63	19.96
Min temperature (°C)	6.70	8.30	8.80	10.75	8.85	10.85
Max temperature (°C)	29.40	30.20	30.25	29.65	28.70	28.90
Average relative humidity (%)	38.69	37.00	43.09	52.55	45.13	53.53
Min relative humidity (%)	9.50	7.50	11.50	16.50	8.00	13.50
Max relative humidity (%)	85.50	97.00	93.00	92.50	94.00	95.50

**Table 6:** Description of the experimental lines.

Line	CODE GB UNSA ^1^
SAL	Salcedo INIA
ACC 23	UNSA-CH-1900023
ACC 24	UNSA-CH-1900024
ACC 25	UNSA-CH-1900025
ACC 26	UNSA-CH-1900026
ACC 27	UNSA-CH-1900027
ACC 28	UNSA-CH-1900028
ACC 29	UNSA-CH-1900029
ACC 30	UNSA-CH-1900030
ACC 31	UNSA-CH-1900031
ACC 32	UNSA-CH-1900032
ACC 33	UNSA-CH-1900033
ACC 34	UNSA-CH-1900034
ACC 35	UNSA-CH-1900035
ACC 36	UNSA-CH-1900036
ACC 37	UNSA-CH-1900037
ACC 38	UNSA-CH-1900038
ACC 39	UNSA-CH-1900039
ACC 40	UNSA-CH-1900040
ACC 41	UNSA-CH-1900041
ACC 42	UNSA-CH-1900042
ACC 43	UNSA-CH-1900043
ACC 46	UNSA-CH-1900046
ACC 47	UNSA-CH-1900047
ACC 49	UNSA-CH-1900049
ACC 50	UNSA-CH-1900050
ACC 52	UNSA-CH-1900052
ACC 53	UNSA-CH-1900053

^1^ CODE GB UNSA: Code assigned by the Project Germplasm Bank of the National University of San Agustin of Arequipa (UNSA).

**Table 7:** Qualitative and quantitative variables utilized for characterizing the 28 quinoa lines.

	Quantitative		Qualitative		
N°	Code	Variable	N°	Code	Variable
1	DFBF	Number of days to floral bud formation (DAS)	1	GH	Growth habit
2	DSF	Number of days to start of flowering (DAS)	2	MSS	Main stem shape
3	D50F	Number of days to 50% flowering (DAS)	3	PPA	Presence of pigmented axils
4	DEF	Number of days to end of flowering (DAS)	4	PRS	Presence of striae
5	DMS	Number of days to milk stage (DAS)	5	PB	Presence of branching
6	DDS	Number of days to doughy grain (DAS)	6	LS	Leaf shape
7	D50PM	Number of days to 50% physiological maturity (DAS)	7	LM	Leaf margin
8	EP	Emergency	8	MS	Male sterility
9	PH	Plant height (cm)	9	DD	Dehiscence degree
10	MSD	Main stem diameter (mm)	10	PD	Panicle density
11	PEL	Petiole length (cm)	11	PS	Panicle shape
12	MLL	Maximum leaf length (cm)	12	PAP	Perigonium appearance
13	MLW	Maximum leaf width (cm)	13	PSY	Pest susceptibility
14	PL	Panicle length (cm)	14	PA	Pericarp aspect
15	PW	Panicle width (cm)	15	EA	Episperm appearance
16	DS	Mildew severity	16	GS	Grain shape
17	SLA	Specific leaf area (cm^2^ g^−1^)	17	SP	Saponin presence
18	NTB	Number of teeth on leaf blade	18	ES	Effusion of saponin
19	GW	Grain width (mm)	19	MSC	Main stem color
20	GT	Grain thickness (mm)	20	STC	Striae color
21	G100W	1000-grain weight (g)	21	PC	Petiole color
22	GHW	Grain hectoliter weight (g cm^−3^)	22	LLC	Leaf lamina color
23	SC	Saponin content (%)	23	LGC	Leaf granules color
24	HI	Harvest index	24	PCF	Panicle color at flowering
25	SY	Grain yield (t ha^−1^)	25	PCPM	Panicle color at physiological maturity
			26	PGC	Perigonium color
			27	PCC	Pericarp color
			28	EC	Episperm color

DAS = days after sowing.

## Data Availability

The data presented in this study are available by request to the corresponding author.
